# Metabolic impairment of non-small cell lung cancers by mitochondrial HSPD1 targeting

**DOI:** 10.1186/s13046-021-02049-8

**Published:** 2021-08-07

**Authors:** Beatrice Parma, Vignesh Ramesh, Paradesi Naidu Gollavilli, Aarif Siddiqui, Luisa Pinna, Annemarie Schwab, Sabine Marschall, Shuman Zhang, Christian Pilarsky, Francesca Napoli, Marco Volante, Sophia Urbanczyk, Dirk Mielenz, Henrik Daa Schrøder, Marc Stemmler, Heiko Wurdak, Paolo Ceppi

**Affiliations:** 1 Interdisciplinary Center for Clinical Research (IZKF), Friedrich-Alexander University of Erlangen-Nuremberg, Erlangen, Germany; 2 Department of Biochemistry and Molecular Biology, University of Southern Denmark, Campusvej 55, 5230 Odense M, Denmark; 3 Department of Surgery, Friedrich-Alexander University of Erlangen- Nuremberg (FAU) and University Hospital of Erlangen, Erlangen, Germany; 4 Department of Oncology At San Luigi Hospital, University of Turin, Orbassano, Turin, Italy; 5 Department of Molecular Immunology, Friedrich-Alexander University of Erlangen-Nuremberg, Erlangen, Germany; 6 Department of Pathology, Odense University Hospital, Odense, Denmark; 7 Department of Experimental Medicine-I, Friedrich-Alexander University of Erlangen-Nuremberg, Erlangen, Germany; 8 Stem Cell and Brain Tumour Group, School of Medicine, University of Leeds, Leeds, LS2 9JT UK

**Keywords:** Non-small cell lung cancer, Metabolism, HSPD1, Targeting, KHS101

## Abstract

**Background:**

The identification of novel targets is of paramount importance to develop more effective drugs and improve the treatment of non-small cell lung cancer (NSCLC), the leading cause of cancer-related deaths worldwide. Since cells alter their metabolic rewiring during tumorigenesis and along cancer progression, targeting key metabolic players and metabolism-associated proteins represents a valuable approach with a high therapeutic potential. Metabolic fitness relies on the functionality of heat shock proteins (HSPs), molecular chaperones that facilitate the correct folding of metabolism enzymes and their assembly in macromolecular structures.

**Methods:**

Gene fitness was determined by bioinformatics analysis from available datasets from genetic screenings. HSPD1 expression was evaluated by immunohistochemistry from formalin-fixed paraffin-embedded tissues from NSCLC patients. Real-time proliferation assays with and without cytotoxicity reagents, colony formation assays and cell cycle analyses were used to monitor growth and drug sensitivity of different NSCLC cells in vitro. In vivo growth was monitored with subcutaneous injections in immune-deficient mice. Cell metabolic activity was analyzed through extracellular metabolic flux analysis. Specific knockouts were introduced by CRISPR/Cas9.

**Results:**

We show heat shock protein family D member 1 (HSPD1 or HSP60) as a survival gene ubiquitously expressed in NSCLC and associated with poor patients’ prognosis. HSPD1 knockdown or its chemical disruption by the small molecule KHS101 induces a drastic breakdown of oxidative phosphorylation, and suppresses cell proliferation both in vitro and in vivo. By combining drug profiling with transcriptomics and through a whole-genome CRISPR/Cas9 screen, we demonstrate that HSPD1-targeted anti-cancer effects are dependent on oxidative phosphorylation and validated molecular determinants of KHS101 sensitivity, in particular, the creatine-transporter SLC6A8 and the subunit of the cytochrome c oxidase complex COX5B.

**Conclusions:**

These results highlight mitochondrial metabolism as an attractive target and HSPD1 as a potential theranostic marker for developing therapies to combat NSCLC.

**Supplementary Information:**

The online version contains supplementary material available at 10.1186/s13046-021-02049-8.

## Background

Lung cancer is the most commonly diagnosed cancer worldwide and the main cause of cancer-related death in both men and women [1]. Non-small cell lung cancer (NSCLC) is the most frequent type occurring in about 85% of cases [2], mainly presenting with an adenocarcinoma (LUAD) or squamous cell lung cancer (SqCC) histology [3]. The standard of care for NSCLC has considerably changed in the last decade with the introduction of target therapies and immunotherapies. These, in synergy with chemo/radiotherapy, allowed to improve the clinical outcome of a fraction of NSCLC patients, in some cases providing a durable disease stabilization [4]. Nevertheless, relapse and occurrence of treatment-resistant phenotypes are still very common, contributing to the current suboptimal scenario of 60 to 70% overall survival for early-stage and between 0 to 10% for patients carrying the advanced disease [5]. This unsatisfactory picture strongly reinforces the need for developing new drugs targeting other fundamental lethal properties of lung cancer cells.

It has long been established that cancer cells alter their metabolism to sustain their increased energetic demands and fuel the malignant phenotype [6]. In contrast, or rather as an expansion of the so-called “Warburg effect”, it is now clear that cancers not only depend on glycolysis for their growth, but also rely on mitochondrial respiration [7], enabling a hybrid or “plastic” behavior in which an opportunistic energetic adaptability determines or facilitate survival and aggressiveness [8]. However, the metabolic alterations frequently found in tumor cells could be turned into therapeutic opportunities, as the newly acquired cancer-specific vulnerabilities or dependencies have been found to represent, in some cases, excellent drug targets [9, 10]. Recent pivotal studies have shown the power of this approach to induce a significant tumor growth arrest [11–13].

Many key cellular metabolic processes are dependent on the functionality of heat shock proteins (HSPs) [14]. HSPs are highly conserved ATP-dependent molecular chaperons mainly involved in maintaining the correct protein structure, for instance avoiding mis-folding or aggregation and protecting cell integrity under stressful conditions [15]. High levels of HSPs have been found in different cancer cells, where they sustain higher metabolic demand and correct misfolded oncoproteins [16]. The mitochondrial heat shock protein family D member 1 (HSPD1), also called HSP60, belongs to this family [17] and together with the co-chaperonin HSP10 plays an essential role in mitochondrial-imported proteins folding or refolding under mitochondrial stress [18]. HSPD1 is involved in several diseases such as neurodegenerative disorders or cardiovascular diseases [19]. In addition, HSPD1 carries a central role in cancer development, with either pro-survival or pro-apoptotic functions reported in different tumor types [20]. In NSCLC, HSPD1 has been proposed as a biomarker, but little is known about its role or the effects of its suppression [21, 22].

Here, we investigated the tumor-promoting role of HSPD1 and explored if its targeting could interfere with the malignant metabolic mechanisms underlying NSCLC. Our results revealed HSPD1 as a ubiquitous lung cancer dependency gene, which maintains the metabolic fitness and promotes growth/survival, thus highlighting it as an attractive therapeutic target for NSCLC.

## Methods

### Cell culture and chemicals

All cell lines were cultured in media supplemented with 10% FBS, 1% Pen/Strep and 1% L-Glutamine (all from Sigma) at 37 °C and 5% CO_2_ in a humidified incubator. H460, H1299, H520, SK-MES-1 and Calu-1 (from ATCC) were cultured in RMPI-1640 (Sigma). H23 and H838 (both from ATCC) cells were cultured in RMPI-1640, supplemented with 1 mM sodium pyruvate (Gibco). A549, 293T, BEAS-2B (ATCC), BEN (DSMZ), murine Lewis lung carcinoma cell line LL2, Ladi2.1 and Ladi3.1 cells (derived from p53fl/fl-LSL KRASG12D/ + mouse NSCLC model) were cultured in DMEM (Sigma). Human cells were STR-profiled, used between passages 3 and 15, examined for mycoplasma regularly (detection kit from Invivogen). The A549-Cas9 cell line was generated by lentiviral transduction with lentiCas9-Blast (Addgene #52962) and selection with 100 μg/mL of blasticidin (Sigma) for 3 days. KHS101 hydrochloride (4888) was purchased from Tocris; cisplatin (CAS 15663–27-1), Z-VAD-FKM (CAS 187389–52-2) and 2-Deoxy-D-Glucose (2-DG, sc-202010) were purchased from Santa Cruz Biotechnology; Necrostatin-1 (N9037-10NMG), Ferrostatin-1 (SML0583-5MG) and 1-Fluoro-2,4-dinitrobenzene (DNFB, D1529) were purchased from Sigma. HB072 was obtained as previously shown [23].

### Proliferation assay and in vitro drug treatment

For in vitro drug treatment, cells were plated in a 96-well plate in low density (5–10% initial confluence) and incubated overnight. On the next day, cells were treated either with vehicle or with the drugs. Plates were loaded into the IncuCyte Zoom (Essen Bioscience) and scanned every 2–4 h. For each scan, phase contrast images were acquired from every well and analyzed by IncuCyte Zoom software. IC_50_ values were calculated after 72 h of treatment as expression of confluency percentage of treated cells normalized to vehicle control (100% confluency) using Graphpad (nonlinear regression curve fitting model) to obtain the values. Proliferation assay was performed as described in [24]. Growth curves were analyzed with IncuCyte Zoom software.

### Cytotoxicity death assay

For cytotoxicity death assay, cells were seeded in 96-well plates in low density (5,000 cells/well) and incubated overnight. 1000X Cytotox Green Reagent or Caspase 3/7 Green Apoptosis Assay Reagent (Essen Bioscience) was diluted in medium and working dilutions of the drug were prepared in Cytotox Green or Caspase3/7 Green supplemented media. After treatment, plates were loaded in IncuCyte Zoom and images were acquired in real-time for phase to quantify growth. Activity of green reagent was simultaneously acquired at the green channel to quantify death. IncuCyte Zoom software was used for the analysis and data export.

### Extracellular flux assays

Oxygen consumption rate (OCR) and extracellular acidification rate (ECAR) measurements were determined using the XFe96 Extracellular Flux Analyzer (Seahorse Bioscience/Agilent Technologies). Cells were seeded in specialized culture microplates at a density of 10,000–20,000 cells/well and prepared as describe in [25]. For Mito Stress Test (Agilent kit 103015–100) 1 μM oligomycin, 1 μM FCCP, 1 μM rotenone and 1 μM antimycin were sequentially injected at regular intervals. OCR, indicator for mitochondrial respiration, and ECAR, indicative of glycolysis, were measured.

### Lentiviral transduction

Plasmids for HSPD1 knock down (TRCN0000029444, TRCN0000029445, TRCN0000029446, TRCN0000029447 and TRCN0000029448 for human cell lines) were purchased from Sigma. Scrambled pLKO.1 (referred to as pLKO) was used as non-targeting control. NLRC5 expression vector (EX-A3335-Lv105) and control vector (Ex-Neg-LV105b) were from GeneCopoeia. Plasmids containing RNAs for COX5B (gRNA sequence #1: ACTTCGCGGAGCTGGAACGC; gRNA sequence #2: CAGCCAAAACCAGACGACGC) and SLC6A8 (gRNA sequence #2: ACGGGGCCGTCGCCCTTGGC; gRNA sequence #3: GCCCTTACCATGCAGACCAG) knock-out (pLENTI-CRISPR-V2) were from Genscript. For production of lentiviral particles, 293 T cells were transfected with 8 μg knock-down/expression vectors and 2 μg of packaging vectors (pMDL, pVsVg and pRevRes) in complex with 24 μg PEI (Polysciences) in 0.9% NaCl. After 48 h, supernatant was collected, centrifuged and filtered. For transduction, 150,000 cells were seeded in a 6-well plate and infected in presence of 8 μg/mL polybrene (Sigma). Selection was done with 3 μg/mL puromycin (Sigma) and cells were maintained in 1 μg/mL puromycin.

### Western blot analysis

For proteins isolation, cells were lysed in RIPA buffer and quantified using Pierce BCA kit (Thermo-Fisher). For cytosolic/mitochondrial fractionation Cytochrome c Release Assay Kit (abcam, ab65311) was used. Proteins lysates (10–20 μg) were resolved on 5%-12% SDS–PAGE gels and transferred to PVDF membrane (Thermo-Fisher). Membranes were blocked in 5% Milk (BioRad) or 5% BSA (Sigma) in 1X TBST and incubated overnight at 4 °C in primary antibodies. Membranes were then washed with 1X TBST and incubated with secondary antibodies (Southern Biotech) for 1 h. The membranes were developed with ECL reagent (Thermo Fisher) on to X-ray films (Thermo-Fisher) using the chemiluminescence imager, AGFA CP100. Rabbit anti-HSPD1 (ab46798, 1:20,000) and rabbit anti-NLRC5 (ab117624, 1:1000) antibodies were purchased from Abcam; mouse anti-TOMM20 (H00009804-M01, 1:1000) was purchased from Abnova; anti-β-Actin (8H10D10) HRP conjugate (1:10,000) was purchased from Cell Signaling. Protein band density was quantified using ImageJ.

### Colony formation assay

To assess clonogenic ability of knockdown cells or KHS101 treated cells, a colony formation assay was performed. Control (pLKO) and knockdown cells were plated in triplicates at a low density (1,500 cells/well) in a 6-well plate and they were allowed to grow until they formed visible colonies. To assess the clonogenic ability of cells upon KHS101 treatment, 200,000 cells were seeded in triplicates in 6-well plates, subsequently treated with 10 μM KHS101 for 5 days, after which media was replaced with normal media, whereas the DMSO cells were collected, counted and seeded in triplicates at low density (1,500 cells/well). Cells were allowed to grow until they formed visible colonies in the controls. Cells were washed with 1X PBS and fixed in 10% (v/v) formalin (Sigma) for 5 min. After washing, colonies were stained with 0.05% crystal violet solution (Biomatik, CAS 548629) for 30 min and washed twice with deionized water. Colonies were photographed and counted.

### FACS analysis

Cell cycle analysis was performed using the Propidium iodide (PI; Sigma) staining. 50,000 cells were seeded in triplicates in 12-well plates and incubated overnight. Samples were prepared as described in [26]. For ROS staining, 50,000 cells were seeded in 12-well plates and the day after they were treated with KHS101 in combination with cisplatin. After 24 h, samples were washed and stained for 30 min at 37 °C with CM-H2DCFDA 5 μM (Thermo Fisher, C6827). Then they were collected in FACS tubes, washed and re-suspended in FACS buffer (2% FBS, 5 mM EDTA in 1X PBS). Samples were run on Cytoflex FACS machine (Beckman) and data were analyzed using FlowJo software v10.6.

### Immunohistochemistry (IHC)

HSP60 (HSPD1) immunohistochemistry was performed on *n* = 30 NSCLC lung surgical specimens using an automated platform (BenchMark, Ventana Medical Systems, Roche, Basel, Switzerland). Briefly, samples were pre-treated for 36 min with antigen retrieval ULTRA CC1 then they were incubated for 32 min at 36 °C with HSP60 (HSPD1) (AbTA800758, Clone OTI3A2, 1:100 dilution, ORIGENE, Rockville, US) primary antibody.

### Immunofluorescence

For mitochondrial staining, 100,000 cells were seeded in 12-well plates. The day after, MitoTracker Green FM (Cell Signaling Technologies, #9074S) was diluted to 400 nM directly into fresh media and added to the cells, which were incubated for 30 min at 37 °C. After incubation, media was changed, and live cells were imaged using IncuCyte-Zoom.

### qPCR

Total RNA was extracted using miRNeasy Mini Kit (Qiagen) according to the manufacturer's instruction. 500 ng of RNA were used to perform reverse transcription with the Tetro cDNA synthesis Kit (Bioline). cDNA was amplified with gene-specific (HK2, FBP1 and PKM) TaqMan probes (Applied Biosystem) using TaqMan Universal Master Mix II (Thermo Fisher Scientific). GAPDH was used as internal control. Quantifications were done by Applied Biosystems 7300 Real Time PCR system and relative mRNA expression level was calculated using the ΔΔCt method.

### CL-100 ProLiFiler screening

26 different lung cancer cell lines (Additional file 1: Table S1) were screened to determine their viability upon treatment with KHS101. The drug was added to the cells one day after seeding and the treatment was performed by nanodrop-dispensing using a Tecan Dispenser. 0.1% DMSO (solvent) and Staurosporine (1,0E-05 M) served as high control (100% viability) and low control (0% viability), respectively. After 72 h of incubation with the compound, cell plates were equilibrated to room temperature for one hour and CellTiter-Glo™ Luminescence Cell Viability Reagent (Promega) was added to the cell suspension. The luminescence was measured 1 h later using a luminometer. IC_50_ values were expressed as percentage of proliferation in presence of solvent alone (100% = high control) as compared to cells treated with 1E-5 M Staurosporine (0% = low control). IC_50_ calculation was performed using GraphPad Prism software with a variable slope sigmoidal response fitting model.

### Generation of CRISPR/Cas9 lentiviral library and CRISPR screen

The CRISPR/Cas9 lentival library was generated as described in [25]. A549 Cas9 cells were transduced with serial dilutions of a virus to find the MOI of ~ 0.3. A549 Cas9 cells were transduced with lentiviral Human GeCKO v2 knockout pooled library part A and part B (a gift from Feng Zhang [27], Addgene # 1000000049) at MOI of 0.3 in the presence of 10 μg/mL of polybrene for 24 h, then replaced the virus medium with fresh growth medium and continued to culture the cells for 48 h. The cells were selected with 50 μg/mL of puromycin for 3 days. Subsequently, 4 million cells were seeded in 10-cm dishes and treated the day after either with vehicle or with 15/10 μM KHS101. A549 Cas9 pLKO were used as positive control. After 2 weeks, the media containing the drug was replaced with normal media to allow the cells to grow again. 40 million cells were then collected for genomic DNA isolation. Next generation sequencing was performed on the Illumina HiSeq 2500 platform in Deep Sequencing Facility of TU Dresden. The raw FASTQ files were analyzed with MAGeCK-VISPR [28].

### Genomic DNA isolation and PCR amplification

Genomic DNA was extracted with NucleoSpin® Blood XL (Machery Nagel # 740,950.50) according to the manufacturer’s protocol. The first round PCR of Next Generation Sequence (NGS) was performed with 26 separate 100 μL redundant reactions, each containing 5 μg of DNA, 50 μL Q5® Hot Start High-Fidelity 2X Master Mix (NEB # M0494L), and 3 μL of a 10 μM solution of each primer (P5 and P7). The PCR was performed as described in [25].

### Transmission electron microscopy (TEM)

A549 cells were treated either with vehicle (DMSO) or KHS101 10 μM for 48 and 96 h. After treatment the cells were fixed with 2% (v/v) glutaraldehyde in 0.04 M phosphate buffer pH 7.4 for 60 min, scraped off, collected in a tube and post-fixed with the same fixative for 24 h. The cells were then washed in PBS and resuspended in 15% BSA for 10 min. The cells were centrifuged, and the cell pellet fixed by slowly adding the fixative for 48 h at 4 °C. The cell pellets were cut in smaller pieces and prepared for EM like following. Pellet fragments were washed in phosphate buffer, stained with 1% osmium tetraoxide in phosphate buffer for 90 min, dehydrated in ethanol series and acetone and embedded in TAAB 812 Embedding Resin (T030, TAAB). Semithin (1 µm) sections were cut on Leica Ultracut UCT microtome and stained with toluidine blue. Ultrathin (70 nm) sections were collected on uncoated nickel grids (M200-NI, Electron Microscope Sciences). The grids were stained with 3% uranyl acetate for 15 min at 60 °C and 3% lead citrate (Leica Ultrostain 2) for 6 min at room temperature. The sections were analyzed with JEM-1400 Plus electron microscope, equipped with Quemsa TEM CCD camera and images obtained using Radius software (TEM Imaging Platform software).

### Survival analysis

Normalized gene expression values for HSPD1 were obtained for lung cancer patient samples from GEO (GSE30219) and mRNA z-score values for the TCGA profile (TCGA LUAD, Cell 2018) from the cbioportal platform [29]. Survival curves were generated using Kaplan–Meier estimate with the samples categorized into ‘low’ and ‘high’ HSPD1 expression groups based on the median of the HSPD1 mRNA expression value. Log-rank test was conducted to obtain the significance between the two groups in R software.

### Differential gene expression analysis and correlation

Differential gene expression (DGE) analysis was performed between the KHS101-sensitive and KHS101-resistant cell lines identified from the drug sensitivity analysis. Briefly, the top four KHS101-sensitive cell lines (NCI-H460, NCI-H1581, LOU-NH91 and A549) and top four KHS101-resistant cell line (SK-LU-1, NCI-H1563, BEN and NCI-H838) were chosen for the analysis. Differential expression between the sensitive and resistant groups was performed using cancer cell line encyclopedia (CCLE) gene expression profile (GSE36133) with RMA log_2_ signal intensity using GEO2R package in GEO with a moderated t-test parameter [30]. 33 up-regulated and 26 down-regulated genes were filtered with a p-value of < 0.05 and log_2_ fold change cutoff of 2. In parallel, Pearson’s correlation analysis was performed between IC_50_ values of KHS101 from 21 cell lines and 18,569 genes expression from CCLE. Based on a correlation value of 0.5 and p-value < 0.05, 298 positively and 97 negatively correlated genes were selected. An overlap analysis was performed with the DGEs list and correlation list to identify a stringent gene list of 12 genes. Correlation analysis between HSPD1 gene expression value obtained from GEO (GSE47206) and KHS101 dose response IC_50_ values was determined using GraphPad Prism 8.

### Differential metabolite analysis

Similar to the differential gene expression analysis, a differential metabolite analysis was performed for the top four KHS101-sensitive and KHS101-resistant group cell lines with the information of 225 log_10_ normalized quantitative metabolites levels across 177 CCLE cell lines [31]. 7 differential metabolites were identified with a log_10_ fold change of 2 and p-value < 0.05.

### Gene ontology analysis

Gene ontology analysis was performed for the top 20 gene candidates identified from the CRISPR knockout screen of A549 cells treated with KHS101. Genes were assessed for the enrichment using GO cellular component 2017b in Enrichr [32].

### Fitness gene and cancer gene dependency

HSPD1 fitness was analyzed as described in [33]. HSPD1 gene’s cancer dependency was analyzed in a panel of NSCLC cell lines (*n* = 52) from PROJECTDRIVE RNAi screen [34]. Using redundant siRNA activity (RSA) gene level metric of threshold -3, the essentiality of the gene in cell lines was determined.

### In vivo experiments

NSG strain (JAX) was used as an experimental model to study the tumor growth of HSPD1 knockdown cells (A549 and H1299). For subcutaneous injections, 0.5 × 10^6^ cells re-suspended in 50 μL 0.9% NaCl were mixed with Matrigel (Corning, 734–0268) in a ratio 1:1 (v:v). Cells were injected in flanks of 9–15-weeks-old NSG with 8 mice per group. Caliper measurements were taken twice a week and tumor volume was calculated using the formula (Length × Width^2^ × π)/6. After 4/5 weeks, mice were euthanized by cervical dislocation and tumors were isolated and weighted.

C57BL/6 strain was used as experimental model to evaluate KHS101 effect on lung metastasis tumor formation. For the tail vein metastasis assay, 0.5 × 10^6^ LL2 cells were re-suspended in 100 μl PBS and injected in the tail vein of female C57BL/6, with 10 mice per group. After 2 weeks either vehicle (5% (v/v) Ethanol-15% (w/v) (2-Hydroxypropyl)-β-cyclo-dextrin (Captisol Technology)) or 6 mg/kg KHS101 (Cellagen Technology) were injected twice a day subcutaneously for 10 days. Lung metastases were monitored by bioluminescence imaging (BLI). Anesthetized mice were intraperitoneally injected with 50 mg/mL D-luciferin (Kayman Chemicals). Bioluminescence images were acquired with Lumina III in vivo Imaging System (IVIS, Perkin Elmer). Time of surrogate survival with disease was calculated as difference between the experimental endpoint and the day of appearance of the bioluminescence signal.

### Statistical analysis

Statistical tests were performed with the GraphPad software (Prism) v.8 comparing groups of different conditions. In all tests at least three biological replicates were used and the statistical significance was considered at p ≤ 0.05.

## Results

### HSPD1 is a fitness gene and a potential target for NSCLC

**Fig. 1:**
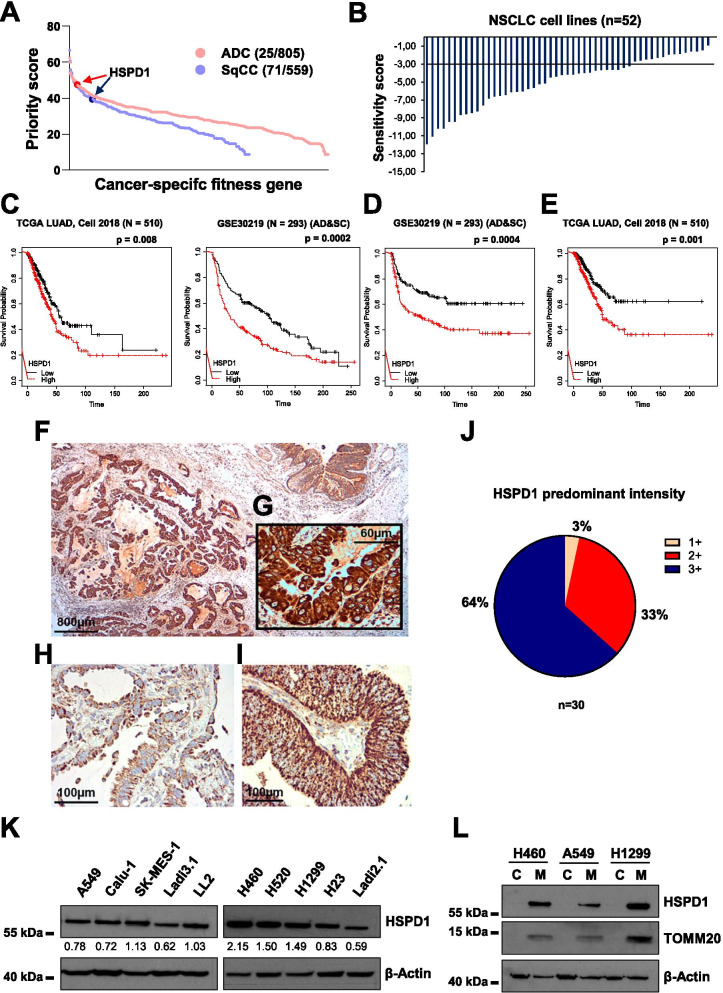
HSPD1 is a fitness gene with prognostic power ubiquitously expressed in NSCLC tumors and cells. **A**) Fitness score for HSPD1 in lung adenocarcinoma (ADC) and squamous cell carcinoma (SqCC). Each dot represents a single gene. **B**) RSA (redundant siRNA activity) waterfall plot of HSPD1 sensitivity score from PROJECTDRIVE showing HSPD1 as an essential gene in non-small cell lung cancer (NSCLC) cell lines (*n* = 52). RSA sensitivity score < -3. **C**) Overall survival analysis between the low- and high-HSPD1 groups in TCGA LUAD (*N* = 510) and GSE30219 (*N* = 293). P-value was calculated using log-rank test. Time is expressed as months. Relapse-free (**D**) and disease-specific (**E**) survival analysis between low- and high-HSPD1 groups in GSE30219 (*N* = 293) and TCGA LUAD (*N* = 510), respectively. P-value was calculated using log-rank test. Time is expressed as months. IHC staining of HSPD1 in cancer tissues (**F**, **G**, **H** and **I**) derived from 30 lung cancer patients (peritumoral bronchial structure in upper right corner of panel **F**). **J**) Pie chart of HSPD1 predominant intensity in NSCLC patient-derived samples. **K**) Western blot quantification of HSPD1 in a panel of different human (A549, Calu-1, SK-MES-1, H460, H520, H1299 and H23) and mouse (Ladi3.1, LL2 and Ladi2.1) NSCLC cells. β-Actin was used as loading control. HSPD1 quantification was normalized to β-Actin. **L**) Western blot analysis of HSPD1 in cytosolic (named as C) or mitochondrial (named as M) fraction. β-Actin was used as loading control and TOMM20 was used as control for cytosolic/mitochondrial fractionation

To understand whether HSPD1 could be an attractive target for NSCLC, we performed a fitness analysis from a previously published dataset of a pan-cancer and genome-wide CRISPR/Cas9 screening of 18,009 genes, which included *n* = 21 adenocarcinoma (ADC) and *n* = 11 squamous cell carcinoma (SqCC) cell lines [35]. As a result, the HSPD1 gene scored high, ranking 25^th^ in ADC and 71^st^ in SqCC cell lines, out of 805 and 559 total ‘fitness’ genes, respectively (Fig. 1A). This initial observation was supported by the results from an independent dataset based on a large-scale RNAi screen of 7,837 genes performed in 52 NSCLC cell lines [34]. HSPD1 was a significant cancer-dependent gene in over 70% of the cells (Fig. 1B, Additional file 1: Table S2), confirming the importance of this gene for NSCLC survival. In addition, survival analysis on HSPD1 mRNA expression showed that the patients with ‘high’ levels of HSPD1 carry a worse prognosis compared to those with ‘low’ HSPD1, in terms of overall (Fig. 1C), relapse-free (Fig. 1D) and disease-specific survival (Fig. 1E). We then investigated the prevalence of HSPD1 expression in tissue samples from a small cohort of NSCLC patients (*n* = 30) by immunohistochemistry (IHC), and observed positive HSPD1 protein expression in 100% of the cases (Fig. 1F-I). The expression pattern was cytoplasmic, granular or diffuse, with some degree of heterogeneity (semi quantitatively scored in a three-level scale), with very high protein levels identified in 64% of specimens (Fig. 1J). No difference was observed between ADC and SqCC samples in terms of HSPD1 expression levels. To confirm HSPD1 expression in NSCLC cell lines grown in vitro, a panel of 10 different cell lines, including three mouse-derived ones (Ladi3.1, LL2 and Ladi2.1) [33], was subjected to western blot quantification. The results indicated detectable HSPD1 protein in all samples (Fig. 1K). Separated protein isolation from mitochondria and cytosolic fractions confirmed that HSPD1 is predominantly localized in the mitochondria in NSCLC cells (Fig. 1L). Therefore, HSPD1 is a mitochondrial protein essential for NSCLC with a strong prognostic value and high prevalence of expression in tissue samples, making it a very valuable candidate for targeted inhibition.

### HSPD1 promotes NSCLC growth in vitro and in vivo

**Fig. 2:**
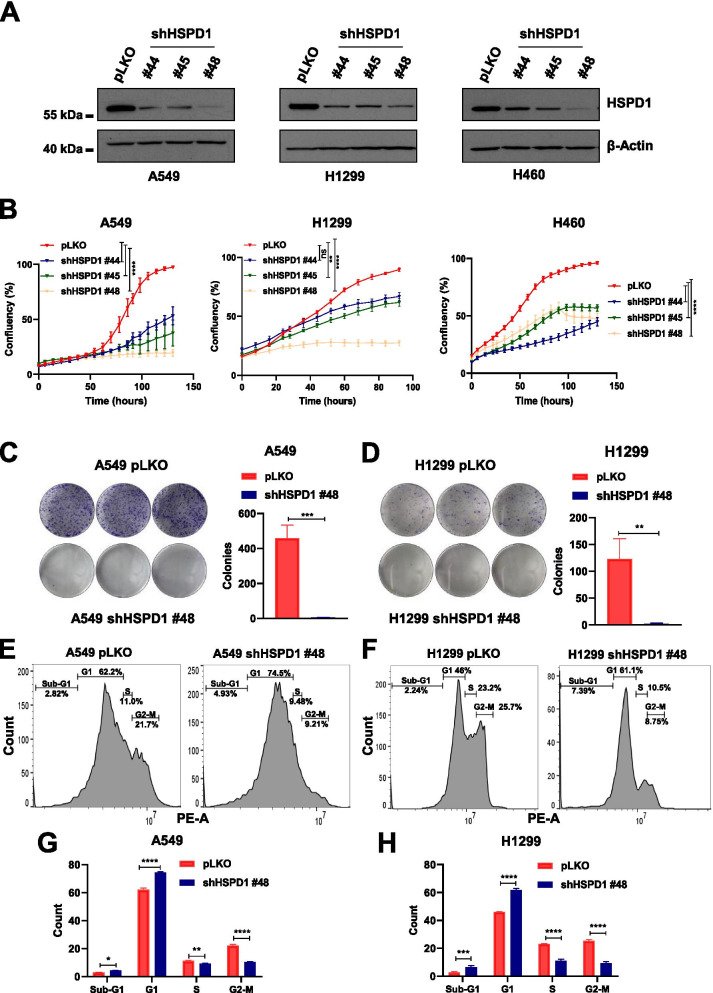
HSPD1 knockdown blocks cell proliferation and clonogenic ability of NSCLC cells. **A**) Western blot analysis of HSPD1 protein levels in A549, H1299 and H460 cells upon infection with 3 independent shRNAs (#44, #45 and #48) targeting HSPD1 compared to scramble-infected cells (pLKO). β-Actin was used as loading control. **B**) Real-time proliferation curves of A549, H1299 and H460 infected with non-targeting pLKO or shHSPD1. The cells’ confluency plotted over time is shown. P-values are from two-way ANOVA. Points are averages of biological replicates ± SD. ** < 0.01, **** < 0.0001. Colony formation of A549 (**C**) and H1299 (**D**) cells with pLKO or shHSPD1, stained with crystal-violet and quantified in triplicates. Bars are average of biological replicates ± SD. P-values are from unpaired *t*-test. ** < 0.01, *** < 0.001. FACS plots of A549 (**E**) and H1299 (**F**) cells infected with pLKO or shHSPD1 and stained with PI for cell cycle analysis. Bar graph showing the % of cells in each cell cycle phase of A549 (**G**) and H1299 (**H**) cells upon infection with pLKO or shHSPD1. P-values are from two-way ANOVA. Bars are average of biological replicates ± SD. * < 0.05, ** < 0.01, *** < 0.001, **** < 0.0001

To study the function of HSPD1 in NSCLC cells in vitro, we analyzed the effects of its knockdown by transducing 5 different NSCLC cell lines with up to 5 independent shRNA sequences (called #44, #45, #46, #47 and #48) (Fig. 2A, Additional file 1: Fig. S1A). Proliferation was evaluated through a real-time cell confluency assay (using the Incucyte system). Shortly after shRNA-based inhibition of HSPD1 expression, the cells showed a decrease in proliferation compared to the controls transduced with a scrambled/non-targeting shRNA, referred to as pLKO (Fig. 2B, Additional file 1: Fig. S1B). In particular, the phenotype was stronger for shRNA sequences providing higher levels of HSPD1 suppression (i.e., #48). HSPD1 knockdown cells were not able to form colonies compared to the control cells when plated at lower density (1,500 cells/well) as observed over two weeks (Fig. 2C-D, Additional file 1: Fig. S1C-D). In line with a strong and durable effect on cell proliferation, a cell cycle analysis (by FACS) showed a significant increase in the percentage of cells in sub-G1 and G1 phases in HSPD1 knockdown cells, in contrast to a reduction in cells residing in S or G2-M phases (Fig. 2E-H, Additional file 1: Fig. S1E-F). To evaluate whether HSPD1 loss-of-function could also affect the growth of normal lung cells, we induced the knockdown in an immortalized non-tumorigenic bronchial epithelial cell line (BEAS-2B). As a result, BEAS-2B cells showed a dramatically reduced growth in vitro (Additional file 1: Fig. S1G-H); however, they were able to completely recover their proliferative capacity as early as one week post infection in contrast to the cancer cells showing a sustained reduction in proliferation capacity (Additional file 1: Fig. S1I).

**Fig. 3:**
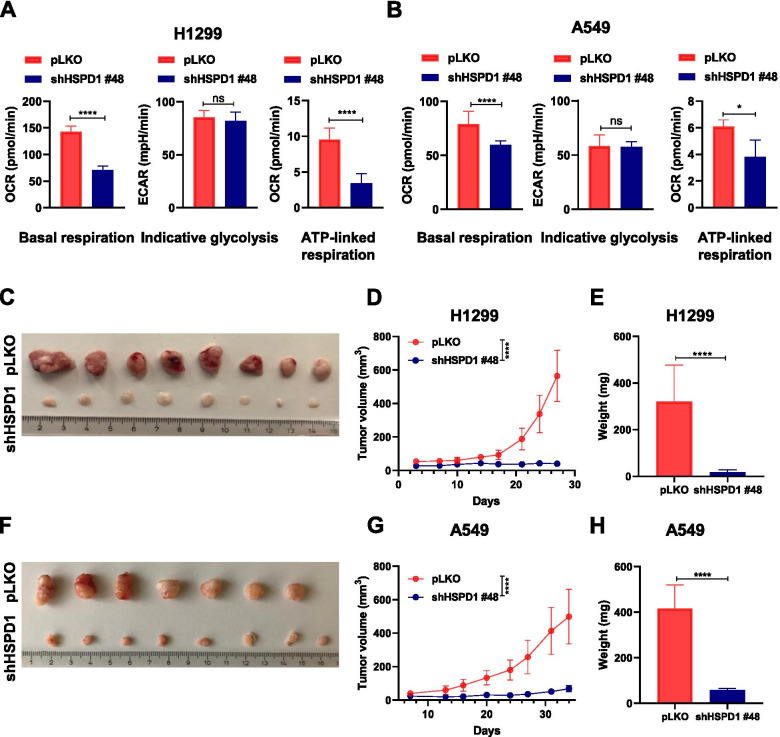
HSPD1 knockdown induces a metabolic impairment and reduces tumor growth in vivo. Quantification of basal respiration, indicative glycolysis and ATP-linked respiration of H1299 (**A**) and A549 (**B**) shHSPD1 cells compared to pLKO cells. Bars are average values of biological replicates ± SD. P-values are from unpaired *t*-test. * < 0.05, **** < 0.0001. Images of tumors from NSG mice injected subcutaneously with H1299 (**C**) or A549 (**F**) pLKO or shHSPD1 cells. Graph showing tumor volume of H1299 (**D**) or A549 (**G**) shHSPD1 cells compared to pLKO cells. P-values are from two-way ANOVA. Points are average of tumors in (**C**, **F**) ± SD. **** < 0.0001. Bar graph showing weight of H1299 (**E**) and A549 (**H**) shHSPD1 tumors compared to pLKO tumors. Bars are average of tumors in (**C**, **F**) ± SD. P-values are from unpaired *t*-test. **** < 0.0001

HSPD1 has a critical function in mitochondrial metabolism [23]. Therefore, to investigate whether the growth suppression observed with HSPD1 knockdown was linked to metabolic alterations, an extracellular metabolic flux analysis was performed using two different cell lines. Throughout the experiments, knockdown cells showed a significant reduction in their basal respiration and in their ATP-linked respiration, suggesting that the alterations in the mitochondrial metabolism may be responsible for the reduction in cell proliferation (Fig. 3A-B). To investigate whether this reduction could be due to a decrease in mitochondrial number, a specific-mitochondrial staining was performed in HSPD1 knockdown cells, which, did not show any difference in the fluorescent intensity of the mitochondrial dye (Additional file 1: Fig. S2A). Finally, we tested whether HSPD1 knockdown inhibited cell growth in vivo*.* To this end, we injected shHSPD1-expressing A549 and H1299 cells subcutaneously in NSG mice to evaluate the tumor growth rate over time. The results markedly indicated that HSPD1 knockdown cells formed very small and slower-growing tumors compared to control pLKO cells, in terms of both tumor volume and tumor weight (Fig. 3C-H), an effect which was maintained for the entire duration of the experiment, hence confirming that HSPD1 is required for NSCLC growth.

### The HSPD1- targeting small molecule KHS101 arrests NSCLC growth

**Fig. 4:**
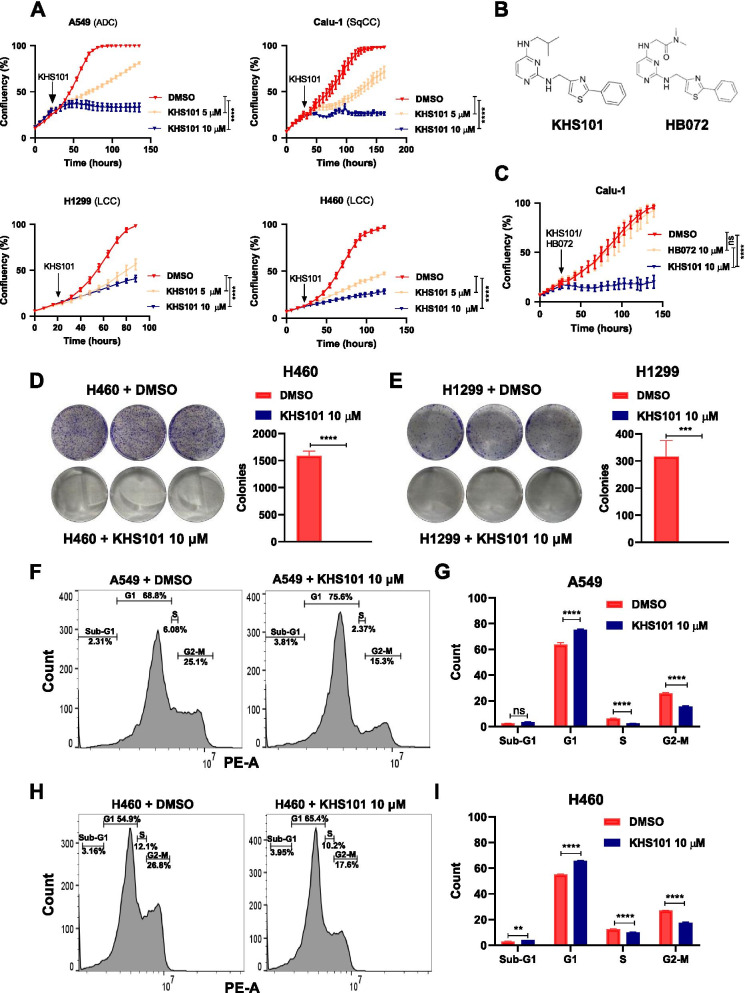
The small HSPD1-targeting molecule KSH101 induces cell growth arrest. **A**) Real-time proliferation curves of A549, Calu-1, H1299 and H460 treated either with vehicle (DMSO) or KHS101. P-values are from two-way ANOVA. Points are average of biological replicates ± SD. **** < 0.0001. **B**) Images of KHS101 and HB072 synthetic molecular structures. **C**) Real-time proliferation curves of Calu-1 cells treated either with KHS101 or with the corresponding inactive KHS101 analog (HB072) compared to vehicle-treated cells. P-values are from two-way ANOVA. Points are average of biological replicates ± SD. **** < 0.0001. Colony formation of H460 (**D**) and H1299 (**E**) treated for 5 days with KHS101 or vehicle and then left until forming visible colonies in drug-free media, then stained with crystal-violet and quantified in triplicates. Bars are average values of biological replicates ± SD. P-values are from unpaired *t*-test. *** < 0.001, **** < 0.0001. FACS plots of A549 (**F**) and H460 (**H**) treated 24 h with KHS101 and stained with PI for cell cycle analysis. Bar graph showing the % of cells in each cell cycle phase of A549 (**G**) and H460 (**I**) cells treated 24 h with vehicle or KHS101. P-values are from two-way ANOVA. Bars are average of biological replicates ± SD. ** < 0.01, **** < 0.0001

KHS101 is a synthetic small molecule reported to impair the growth of glioblastoma cells in vitro and in vivo via inhibition of HSPD1 chaperone activity [23]. As KHS101 leads to metabolic exhaustion and apoptosis in glioblastoma cells, we first tested as to whether the compound elicits cytotoxicity in NSCLC cell lines. After treatment with KHS101, 6 human NSCLC cell lines showed a significant reduction in cell proliferation that was dependent on the compound concentration (Fig. 4A, Additional file 1: Fig. S2B-C). To verify the specificity of HSPD1 targeting, we exposed the cells to HB072, an HSPD1 non-targeting chemical analogue of KHS101 [23] (Fig. 4B), which did not significantly affect cell growth (Fig. 4C, Additional file 1: Fig. S3A). After 5 days of continuous drug treatment, NSCLC cells lost their clonogenic growth ability, as shown in a colony formation assay (Fig. 4D-E, Additional file 1: Fig. S3B). In line with HSPD1 knockdown experiments, cell cycle analysis showed a significant reduction in the percentage of cells in S and G2-M phases concomitant with an increase of cells in G1 phase (Fig. 4F-I), as assessed 24 h after KHS101 treatment. Finally, since many conventional anti-cancer therapeutic strategies fail to target non-proliferating cancer cells, we sought to test whether the KHS101-induced growth inhibitory effect was maintained altering the proliferative state of the cells in a controlled fashion. Therefore, after being synchronized overnight using a low FBS concentration (0.5%), the cells were treated with KHS101 in presence of media with increasing FBS, ranging from 0 to 5%. As a result, no differences were measured in KHS101 efficacy in the different conditions by a real-time proliferation assay (Additional file 1: Fig. S3C), indicating that KHS101 is effective independently of the cells’ proliferative status. In conclusion, these data indicate that chemical targeting of HSPD1 markedly reduces the growth of NSCLC cell lines.

### KHS101 induces metabolic disruption in NSCLC cells leading to cell death

**Fig. 5:**
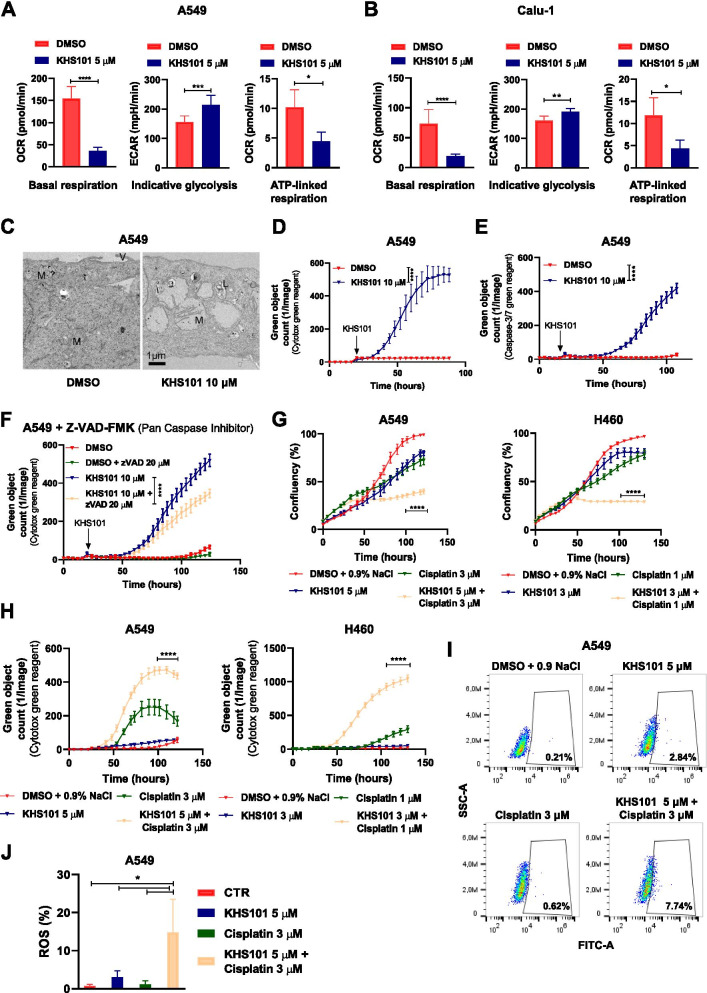
KHS101 induces a metabolic impairment and death in NSCLC cells. Bar graphs showing quantification of basal respiration, indicative glycolysis and ATP-linked respiration in A549 (**A**) or Calu-1 (**B**) treated with KHS101 for 24 h compared to control cells (DMSO). Bars are average values of biological replicates ± SD. P-values are from unpaired *t*-test. * < 0.05, ** < 0.01, *** < 0.001, **** < 0.0001. **C**) Electron microscopy images of A549 treated 96 h either with vehicle (DMSO) or KHS101 10 μM (M: mitochondria, V: microvilli). Dead cell quantification as green object count using Cytotox green reagent (**D**) or caspse-3/7 reagent (**E**) of vehicle or KHS101 treated A549 cells. **F**) Dead cell quantification as green object count of A549 treated either with DMSO or KHS101 in combination with pan-caspase inhibitor Z-VAD-FMK. In **D**, **E** and **F** points are average of biological replicates ± SD. P-value are from two-way ANOVA. **** < 0.0001. **G**) Real-time proliferation curves of A549 and H460 treated with KHS101 and/or cisplatin compared to control cells. Cell death is shown as green object count in **H**). Points are average values of biological replicates ± SD. P-values are from two-way ANOVA and analyzed comparing the combination of drugs to control and the respective drug alone. **** < 0.0001. Percentage of ROS-positive cells in A549 cells treated with KHS101 and/or cisplatin for 24 h are shown in FACS plots (**I**) and a graph bar (**J**). P-values are from one-way ANOVA. Bars are average of biological replicates ± SD. * < 0.05

To test whether KHS101 affects energy metabolism in NSCLC cell lines, we performed an extracellular flux analysis 24 h after KHS101 treatment. The results indicated a strong reduction in cellular basal respiration and in ATP-coupled respiration capacity. Concomitantly, a significant increase was observed for basal glycolysis (Fig. 5A-B). We next asked as to how fast KHS101 would induce such alterations and treated the cells for shorter periods (ranging from 0.5 h to 6 h), observing that glycolytic activity increases simultaneously with oxidative phosphorylation (OXPHOS) reduction from less than one hour after treatment (Additional file 1: Fig. S4A-B), and suggesting that the metabolic rewiring induced by KHS101 is an early event, as previously described [23]. We further investigated metabolic alterations evaluating the expression of some key metabolic enzymes in A549 HSPD1 knockdown cells and A549 cells treated with KHS101 for 72 h. We observed a significant increase in hexokinase 2 (HK2) expression (Additional file 1: Fig. S4C), which was consistent with the detected increase in glycolysis. In addition to metabolic flux phenograms, cell morphology was found altered in cells exposed to 10 µM KHS101. Treated cells presented an altered ultrastructure and, after 96 h, severe swelling of mitochondria and sparse presence of cristae was observed. Moreover, an increased number of secondary lysosomes was found indicating a marked cellular degeneration associated with a loss of microvilli (Fig. 5C). These alterations were already detectable as early as 48 h after treatment (Additional file 1: Fig. S4D). As a result of these alterations, and differently to HPSD1 knockdown, KHS101 treatment induced cell death, as indicated by the incorporation of fluorescent Cytotox green dye (Fig. 5D, Additional file 1: Fig. S4E). To test whether the affected cells were undergoing apoptosis, we performed a caspase 3/7 activity assay, which showed specific activation of apoptosis 2 days after the treatment (Fig. 5E, Additional file 1: Fig. S4F-G). Co-administration of the pan-caspase inhibitor Z-VAD-FMK could efficiently suppress this caspases activation (Additional file 1: Fig. S4I) but did not rescue the cells from KHS101 cytotoxicity (Fig. 5F, Additional file 1: Fig. S4H), thus indicating that cell death is not occurring solely as a result of apoptotic induction. Interestingly, no protection was observed also in presence of necroptosis or ferroptosis inhibitors (Additional file 1: Fig. S4J-K), suggesting that the metabolic alterations caused by KHS101 could not be rescued by blocking cell death pathways. Furthermore, to investigate the impact of KHS101 in vivo*,* we tested the sensitivity of the tumor-forming mouse-derived NSCLC cell line LL2 (Additional file 1: Fig. S5A-B), and subsequently injected them in the tail vein of BL6 mice to evaluate the growth of lung metastasis upon KHS101 treatment. Lung tumors-bearing mice that were treated subcutaneously for 2 weeks with 6 mg/kg KHS101 (twice a day) did not show a significant difference in tumor area, as evaluated by IVIS measurement. However, KHS101-treated mice showed a prolonged disease-specific survival time (Additional file 1: Fig. S5C), in line with a non-significant trend for a reduced number of lung lesions, as macroscopically detected (Additional file 1: Fig. S5D).

Finally, we investigated the in vitro effects of KHS101 in combination with cisplatin, one of the most commonly used chemotherapeutic drug used in relapsing NSCLC [36]. Strikingly, a low cisplatin dose (1 or 3 µM) that alone was not able to strongly impair cell growth or survival, showed to induce a marked growth suppression (Fig. 5G, Additional file 1: Fig. S6A) and increase in cell death (Fig. 5H, Additional file 1: Fig. S6B) when combined with KHS101 (also at sub-lethal doses), resulting in a 2 to 4-fold reduced relative growth compared to cisplatin alone in three different cell lines (Additional file 1: Fig. S6C). Cisplatin exerts its activity via upregulation of reactive oxygen species (ROS) [37] and by fluorescent ROS staining of treated cells we found that the combination of cisplatin and KHS101 synergistically increased ROS activation (Fig. 5I-J). These data suggest that HSPD1 targeting can also induce cell death and increase the efficacy of chemotherapeutic treatments.

### Sensitivity to KHS101 is related to the metabolic state of the cells

**Fig. 6:**
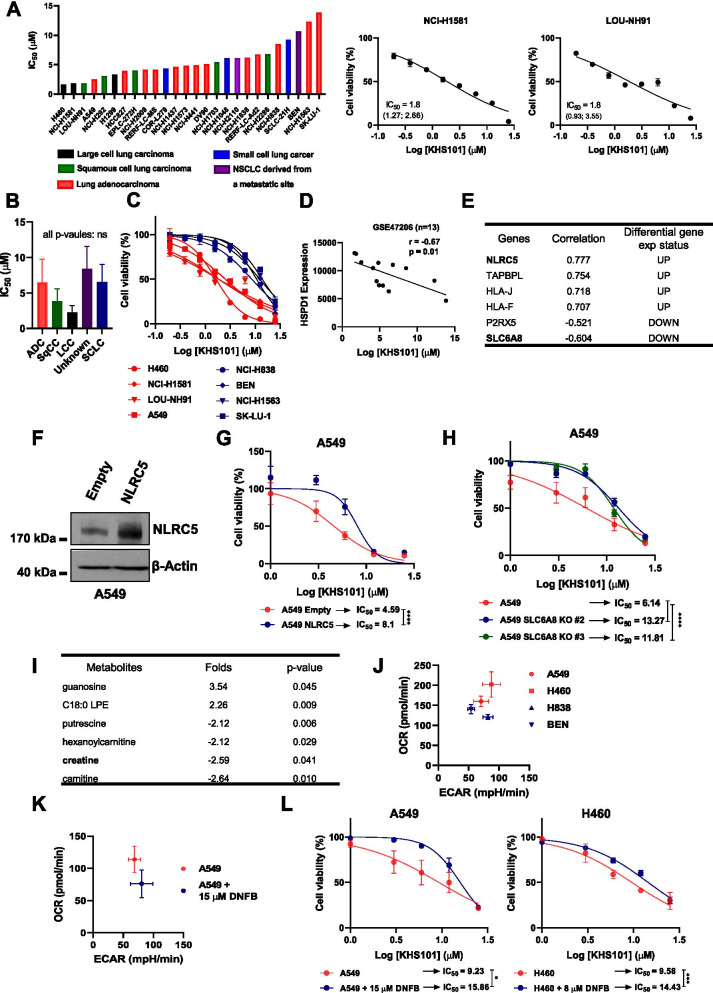
KHS101 sensitivity is related to NSCLC metabolic state. **A**) Bar graph showing IC_50_ values (μM) of all the 26 NSCLC cells belonging to CL-100 ProLiFiler screening and (on the right panel) dose–response curves to KHS101 treatment of the indicated two cell lines. Points are average values of replicates ± SD. IC_50_ values (μM) are shown with 95% confidence intervals. **B**) Bar graph showing the average IC_50_ for each NSCLC histotype and SCLC. Bars are average ± SD. P-values are from one-way ANOVA. **C**) Dose–response curves of the 4 most sensitive cell lines (H460, NCI-H1581, LOU-NH91, A549) compared to the 4 most resistant ones (NCI-H838, BEN, NCI-H1563, SK-LU-1) resulting from the sensitivity screening. Points are average of biological replicates ± SD. **D**) Correlation between HSPD1 gene expression and KHS101 IC_50_ values in a panel of NSCLC cell lines from GSE47206 (*n* = 13). **E**) Genes with most differential expression between KHS101-sensitive and KHS101-resistant groups (log_2_ fold change cut-off of 4 and p-value < 0.05). **F**) Western blot analysis of NLRC5 protein level in A549 cells overexpressing NLRC5 or empty vector. β-Actin was used as loading control. **G**) Dose–response curves of A549 overexpressing NLRC5 compared to empty control cells. **H**) Dose–response curves of A549 SLC6A8 KO compared to control cells. **I**) Metabolite analysis between KHS101-sensitive and KHS101-resistant cells with a log_10_ fold change of 2 and p-value < 0.05. **J**) Metabolic phenogram showing OCR (basal respiration) and ECAR (indicative glycolysis) values of creatine high (H460, A549) and creatine low (BEN, H838) cells. **K**) Metabolic phenogram showing OCR and ECAR levels in A549 treated for 24 h with creatine-kinase inhibitor (DNFB). **L**) Dose–response curves of A549 and H460 cells in presence of creatine-kinase inhibitor DNFB. In **G**, **H** and **L** IC_50_ values (μM) are shown. P-values are from two-way ANOVA and points are average of biological replicates ± SD. * < 0.05, *** < 0.001, **** < 0.0001. All dose–response curves are normalized to the DMSO control

We further profiled in vitro sensitivity to KHS101 by performing a screening on 26 different lung cancer cell lines, measuring cell viability 72 h after treatment. Interestingly, none of the cell lines in the panel appeared to be completely resistant (Fig. 6A, Additional file 1: Fig. S7A). KHS101-induced cytotoxicity was independent of the lung cancer type (NSCLC or SCLC) or NSCLC histological subtype (Fig. 6B). However, cells showed different grades of sensitivity towards the compound with IC_50_ values ranging from 2 to 14 μM (Fig. 6C, Additional file 1: Fig. S7A). These results were independently validated comparing KHS101 sensitivity in the most sensitive and resistant cell lines based on percentage of inhibition of cell proliferation (Additional file 1: Fig. S8A). To find possible determinants of drug resistance, cell lines with different genetic alterations were compared, but no particular genetic background was found as significantly protective (Additional file 1: Table S3). To identify possible molecular determinants of drug sensitivity, we performed a transcriptomic analysis on cell lines based on their IC_50_ values. HSPD1 mRNA expression correlated with IC_50_ values obtained from the screening (Fig. 6D). In order to have a stringent and genome-wide comparison, we used a 2-sided statistical approach. First, the 4 most resistant (NCI-H838, BEN, NCI-H1563 and SK-LU-1) and the 4 most sensitive NSCLC cell lines (H460, NCI-H1581, LOU-NH91 and A549) were grouped and the differentially expressed genes between the two groups were identified and ranked according to their fold differences (Additional file 1: Table S4-5). Moreover, using available data from all the cells in the screening, we correlated the expression of each gene with the KHS101 IC_50_ values and ranked them based on Pearson metrics (Additional file 1: Table S6-7). The results from these two analyses were overlapped (Additional file 1: Fig. S8B), obtaining a list of potential up- and down- regulated genes in cells resistant to KHS101 (Fig. 6E). The top up-regulated resistance-conferring gene was the transcription factor NLRC5, which acts as a transactivator for the MHC class I genes [38] (some of which were also up-regulated in the analysis, like HLA-J or HLA-F). To validate this finding, we lentivirally overexpressed NLRC5 in sensitive NSCLC cells (Fig. 6F), and detected a significantly decreased cytotoxic effect of KHS101, indicating cellular resistance (Fig. 6G, Additional file 1: Fig. S8C). Among the genes down-regulated in the resistant cell lines, the top hit was SLC6A8 (Fig. 6E), a sodium chloride-dependent creatine transporter 1 [39]. This evidence was validated by introducing a specific CRISPR/Cas9-based knock-out of SLC6A8, which increased cells viability of sensitive cell lines treated with KHS101 (Fig. 6H). Since SLC6A8 is a creatine transporter we speculated that it could alter KHS101 sensitivity by changing the metabolic features of the cells. Analysis of a previously published metabolome profiling of NSCLC cell lines [31] revealed that cells that we determined to be the most KHS101 sensitive had a higher creatine level (Fig. 6I), which was consistent with the results from the transcriptomic analysis (i.e. higher expression of the importer) suggesting a possible effect of creatine metabolism in the response to KHS101. Creatine can be phosphorylated by the cytosolic creatine kinase (C-CK) or by the mitochondrial isoenzymes (MtCK) to provide a rapid source of ATP [40] thereby fueling ATP-dependent cellular processes. To test whether differences in cellular creatine levels could be correlated with the cells’ metabolic state, we performed extracellular flux analysis of cells with higher (A549 and H460) and lower creatine (H838 and BEN) baseline levels [31] and found that cells with higher creatine had higher basal respiration (Fig. 6J). To prove this correlation further functionally, we inhibited creatine kinase activity with the specific inhibitor DNFB which decreased the basal respiration level of A549 cells (Fig. 6K), concomitantly increasing the cells’ resistance to KHS101 as assessed by cell viability assays (Fig. 6L). Taken together these findings suggest that a higher dependency on OXPHOS due to the alteration of creatine metabolism activity could determine the sensitivity to KHS101 of NSCLC cells.

### KHS101 sensitivity depends on OXPHOS activity

**Fig. 7:**
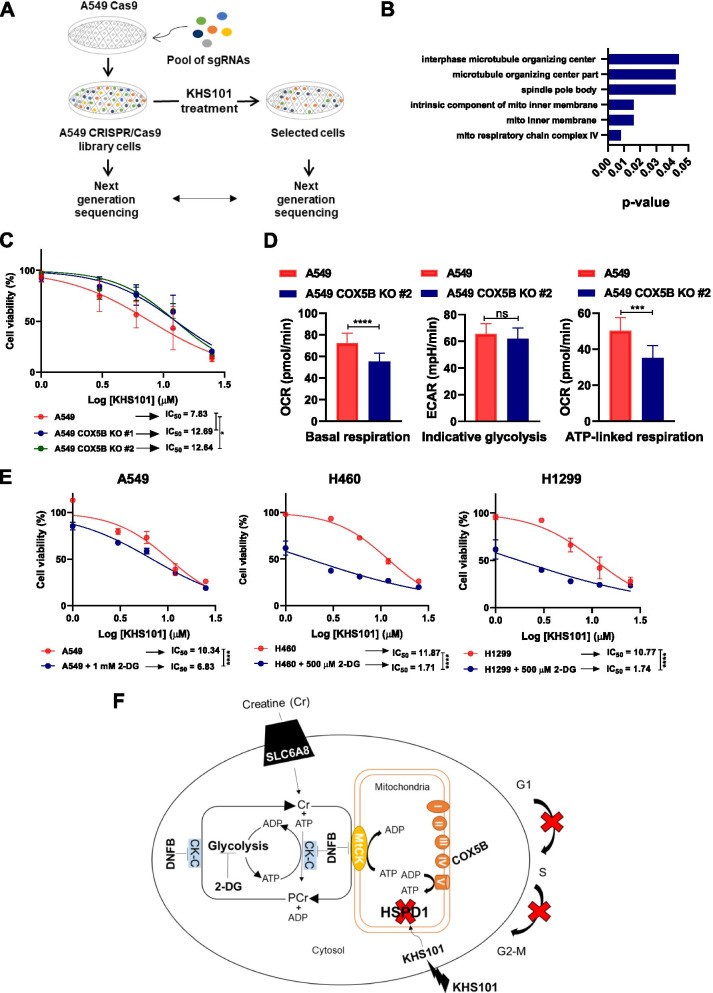
Mitochondrial metabolism influences NSCLC cells response to KHS101. **A**) Scheme showing the genome-wide CRISPR-Cas9 screening platform employed to identify KHS101 resistance genes. **B**) Gene ontology (GO) analysis of the top 20 candidates identified from the knockout screen in cells after treatment with KHS101. GO was performed with the cellular component 2017b gene set library in Enrichr tool. **C**) Dose–response curves to KHS101 (normalized to DMSO control) of A549 cells overexpressing two gRNAs to knockout COX5B, compared to parental A549. Points are averages of replicates ± SD. IC_50_ values (μM) are shown. P-values are from two-way ANOVA. * < 0.05. **D**) Bar graphs showing quantification of basal respiration, indicative glycolysis and ATP-linked respiration of A549 COX5B knockout cells compared to parental cells. Bars are average of replicates ± SD. P-values are from unpaired *t*-test. *** < 0.001, **** < 0.0001. **E**) Dose–response curves (normalized to DMSO control) of A549, H460 and H1299 cells in presence of glycolysis inhibitor 2-deoxy-D-glucose (2-DG). Points are average values of replicates ± SD. IC_50_ values (μM) are shown. P-values are from two-way ANOVA. **** < 0.0001. **F**) Schematic representation of the metabolic alterations that occur in NSCLC cells upon HSPD1 loss

To further investigate KHS101 resistance at a broader level, we performed a genome-wide CRISPR/Cas9-based dropout screening. A genetically barcoded whole-genome library (GeCKO v2) was transduced in A549 cells overexpressing the Cas9 nuclease. These cells were treated with two different KHS101 concentrations (10 and 15 μM) or DMSO as control (Fig. 7A). None of the cells were able to survive the treatment with the 15 μM KHS101 dose, suggesting that is not possible to develop a complete resistance to this compound by the elimination of a single gene. By contrast, we could observe non-growing and morphologically altered cells surviving the 10 μM dose. After 2 weeks of survival (after all control pLKO-infected Cas9-A549 cells treated with the same KHS101 dose underwent complete cell death), the drug treatment was stopped to allow the selected cells to grow and be processed for the DNA preparation and the next-generation sequencing steps. The sequencing results on untreated cells indicated that it was possible to detect a total of 116,700 sgRNAs (all gRNAs excluding those with no counts) present in the library, with a 97% representation, a level similar to previous screenings [25], while in the treated group this percentage decreased to 56%, as a result of the drug selection. ‘Hits’ were ranked and gene ontology analysis on the 20 highest ranking ‘hits’ (Additional file 1: Table S8) indicated that the genes whose knockout increased cellular resistance to KHS101 were mainly involved in regulating mitochondrial structure and activity (Fig. 7B). The COX5B gene, a subunit of the cytochrome c oxidase, was the ‘top hit’. In order to independently validate this finding, we knocked out COX5B by two independent gRNAs in A549 cells, and in both cases, we found the emergence of a more KHS101-resistant phenotype (Fig. 7C). COX5B knock-out cells presented a reduction in their basal respiration and ATP-linked respiration (Fig. 7D), strengthening the notion that KHS101 sensitivity is linked with cellular oxidative activity, in line with the results obtained from creatine metabolism. Conversely, blocking glycolysis by 2-deoxy-D-glucose (2-DG) treatment significantly enhanced KHS101 sensitivity in different cell lines (Fig. 7E), further confirming that cancer cells respond to KHS101 proportionally to their OXPHOS-dependence. Therefore, HSPD1 targeting induces a mitochondrial metabolic breakdown in NSCLC, which results in loss of growth potential and cell death, and the small molecule KHS101 is particularly active in OXPHOS-dependent cells.

## Discussion

The identification of novel targets and the development of more effective drugs is of paramount importance for improving the clinical management of NSCLC. Metabolism has recently gained much attention in the oncological field and anti-metabolism strategies have been proposed to reduce the lethal effects of NSCLC [41–43]. We report here heat shock protein HSPD1 as a theranostic metabolic marker for NSCLC, whose elimination can have profound effect on outcome of the patients.

Heat shock proteins (HSPs) are molecular chaperones categorized according to their molecular sizes into small HSPs and large HSPs [16]. HSPs can be highly expressed in cancer cells, where they promote cell proliferation, metastasis and drug resistance. HSPD1 is an ATP-dependent HSP mainly localized in the mitochondria, playing an essential role in guaranteeing the correct folding of the mitochondrial-imported proteins [44] and some data are available on its role as a stemness/metastasis regulator in other cancers [22, 45, 46]. In lung cancer, *HSPD1* has been previously found highly expressed in NSCLC tissues [47] and identified as a predictive marker for survival in both smokers and non-smokers patients [48]. Our findings confirm that HSPD1 is ubiquitously expressed at a high level (as detectable by IHC in all samples) and is associated with adverse prognosis, adding the notion that its expression is essential for NSCLC survival (fitness gene), making it a very attractive target for future therapy. In order to more functionally explore this possibility, we evaluated the effect of HSPD1 targeting achieved by either shRNA-mediated knockdown or by a specific small molecule. In shRNA experiments, the level of growth suppression measured for each shRNA sequence was generally proportional to the degree of HSPD1 knockdown. Importantly, even if a complete protein elimination was not achieved, the cell growth blockage obtained by the best performing shRNA (#48) was durable and very effective both in vitro and in vivo in all the cell lines investigated, suggesting that this behavior is not correlated to NSCLC histotypes or HSPD1 expression level. When cellular metabolic changes were measured, cells with HSPD1 knockdown presented a strong reduction in basal respiration and impaired capacity in synthesizing ATP though OXPHOS, in line with the role of HSPD1 in the (re)folding of mitochondrial-imported oxidative respiration proteins [49]. Noteworthy, while this investigation mainly focused on the effects on metabolism, other functions of HSPD1 might have contributed to the loss of viability observed upon its knockdown and could be further investigated. For example, HSPD1 can act as immunomodulant [50], can stimulate phagocytosis of senescent cells [51], and regulate apoptosis and intracellular trafficking [52, 53]. In any case, our data clearly indicate the activity of HSPD1 as essential for maintaining NSCLC metabolic fitness and its loss causes a profound energetic breakdown affecting the ability of the cancer cells to divide and expand, making it an attractive therapeutic target.

An emerging way of chemically disrupting the functionality of HSPD1 is the treatment with the synthetic small compound KHS101. This compound was originally identified through a phenotypical screen as an inducer of neuronal differentiation both in vitro and in vivo [54]. KHS101 promotes HSPD1 aggregation thereby altering the metabolic activity of glioblastoma cells [23]. In NSCLC, we observed that KHS101-treated cells were very severely growth arrested, in analogy to what was observed with the HSPD1 knockdown approach. OXPHOS activity was also efficiently repressed, even with low micromolar KHS101 concentrations. In contrast to HSPD1 knockdown, KHS101-treated cells increased glycolysis, suggesting that they might try to compensate for the loss of ATP production. This increase is, however, not sufficient for the cells to gain resistance and survive the treatment. Indeed, KHS101-treated NSCLC cells underwent significant cell death, probably due to the formation of irreversible HSPD1 multi-aggregates coagulated by KHS101, which can disrupt the energetic capacity of the cancer cells, as previously shown [23]. Notably, co-administration of specific inhibitors of death pathways did not rescue KHS101 treated cells, indicating that the occurring death is strong, multifactorial, and irreversible, all features of crucial importance for a novel anti-cancer therapeutic strategy.

In order to better understand the biology of HSPD1 targeting, two independent unbiased screenings were carried out: a differential gene expression analysis on KHS101 sensitive and resistant cells and a genome-wide CRISPR/Cas9-based dropout screening on drug treated cells. The first approach identified a biomarker of increased sensitivity, the creatine importer SLC6A8, which led us to discover OXPHOS dependency as condition for better KHS101 efficacy. Moreover, a potential role in determining KHS101 resistance of the MHC class I genes transactivator NLRC5 has been here identified. Based on its importance on inflammatory processes in tumors [31], the molecular details of this link should be in the future determined. The second approach came independently to a similar and converging conclusion, by identifying mitochondrial metabolism genes, and in particular the subunit of the cytochrome c oxidase complex (COX5B), as enriched in cells surviving KHS101. This evidence highlights the targeting of mitochondrial metabolism as an effective therapeutic strategy to achieve a complete, irreversible elimination of NSCLC.

The occurrence of drug-overcoming mutations is a typical feature of NSCLC, which has unfortunately so far limited the success of targeted therapies [55]. It is noteworthy that not a single cell from the whole-genome CRISPR/Cas9 library was able to survive the KHS101 selection when administered at the highest dose (15 μM). At the lower dose (10 μM), a small population of cells was able to persist, but could not grow (and be processed for sequencing) until the drug was entirely removed from the experiment. This is a very important observation in line with the observed inability of death pathways inhibitors to protect in vitro from KHS101, and in line with the data from the two independent fitness analyses on *HSPD1* gene and with the drug screening from large collections of NSCLC cell lines of various genetic backgrounds, which confirmed that no single genetic alteration can guarantee a complete protection from the treatment with KHS101. The sensitivity towards KHS101 is also independent from the lung cancer type or NSCLC histotype, suggesting that a HSPD1 targeting approach might be valuable also for other cancer types. Another aspect of fundamental importance to consider for NSCLC drug development is the potential successful combination with chemotherapy, as chemotherapy still represents the backbone of therapeutic regimens, especially in late-stage lung cancer patients [56–58]. Even if the data here presented will require future dedicated experimentation, the results are encouraging, as KHS101 acted synergistically with cisplatin in vitro, enhancing the production of reactive oxygen species and reducing the dose of cisplatin needed to slow down or kill the cancer cells. These observations reinforce the potential relevance of designing and developing HSPD1-targeting drugs for NSCLC therapy. This goal, however, is not likely to be achieved by KHS101 itself, which this study found active at relatively high concentrations. KHS101 therefore stands out as a rather excellent tool compound for accelerating discoveries for HSPD1 targeting, but novel drugs (or modifications of the same compound) with improved affinity will need to be designed and tested, for instance by taking advantage of emerging targeting techniques based on induction of selective intracellular proteolysis [59, 60]. The activity of these drugs could be in the future tested beyond NSCLC, as HSPD1 appears to be a fundamental oncoprotein also in other cellular contexts [45, 46].

## Conclusions

In conclusion, we showed that HSPD1 elimination interferes with NSCLC metabolic activity causing a strong OXPHOS-dependent energetic impairment, which the cancer cells fail to overcome, highlighting HSPD1 as a powerful theranostic marker for improving lung cancer therapy.

## Supplementary Information

**Additional file 1.**

## Data Availability

The datasets used and/or analyzed during the current study are available from the corresponding author on reasonable request.

## Abbreviations

NSCLC: Non-small cell lung cancer
HSPs: Heat shock proteins
ADC: Adenocarcinoma
SqCC: Squamous cell lung cancer
OCR: Oxygen consumption rate
ECAR: Extracellular acidification rate
DGE: Differential gene expression
IHC: Immunohistochemistry
HSPD1: Mitochondrial heat shock protein family D member 1
NLRC5: NOD-Like Receptor C5
SLC6A8: Solute Carrier Family 6 Member 8
COX5B: Cytochrome C Oxidase Subunit 5B
DNFB: 1-Fluoro-2,4-dinitrobenzene
2-DG: 2-Deoxy-D-Glucose
