# Supplementary material for: Metabolic impairment of non-small cell lung cancers by mitochondrial HSPD1 targeting
Source: J Exp Clin Cancer Res. 2021 Aug 7;40:248. doi: 10.1186/s13046-021-02049-8 (PMC8348813; doi:10.1186/s13046-021-02049-8)
Supplement: Supplementary file 1 — Additional file 1. [file 13046_2021_2049_MOESM1_ESM.docx]

**SUPPLEMENTARY INFORMATION**

**ADDITIONAL FILE 1**

**Figure S1** A) Western blot analysis of HSPD1 protein level in SK-MES-1 and Calu-1 cells upon infection with respectively 5 or 3 independent shRNAs (#44, #45 #46, #47 and #48) targeting HSPD1 compared to scramble-infected cells (pLKO). β -Actin was used as loading control. B) Real-time proliferation curves of SK-MES-1 and Calu-1 infected with non-targeting pLKO or shHSPD1. Plotted is cells’ confluency over time. P-values are from two-way ANOVA. Points are average of replicates ± SD. ****<0.0001. Colony formation of H460 (C) and Calu-1 (D) cells infected with pLKO or shHSPD1, stained with crystal-violet and quantified in triplicates. Bars are average of biological replicates ± SD. P-values are from unpaired *t*-test. **<0.01, ***<0.001. E) FACS plots of Calu-1 cells infected with pLKO or shHSPD1 and stained with PI for cell cycle analysis. F) Bar graph showing the % of cells in each cell cycle phase of Calu-1 cells upon infection with pLKO or shHSPD1. P-values are from two-way ANOVA. Bars are average values of replicates ± SD. *<0.05, ****<0.0001. G) Western blot analysis of HSPD1 protein level in BEAS-2B cells upon infection with one shRNA (#48) targeting HSPD1 compared to scramble-infected cells (pLKO). β -Actin was used as loading control. H) Real-time proliferation curves of BEAS-2B infected with non-targeting pLKO or shHSPD1. P-value is from two-way ANOVA. Points are average values of replicates ± SD. ****<0.0001. I) Real-time proliferation curves of A549, H1299 and BEAS-2B infected with non-targeting pLKO or shHSPD1 after two weeks from infection.


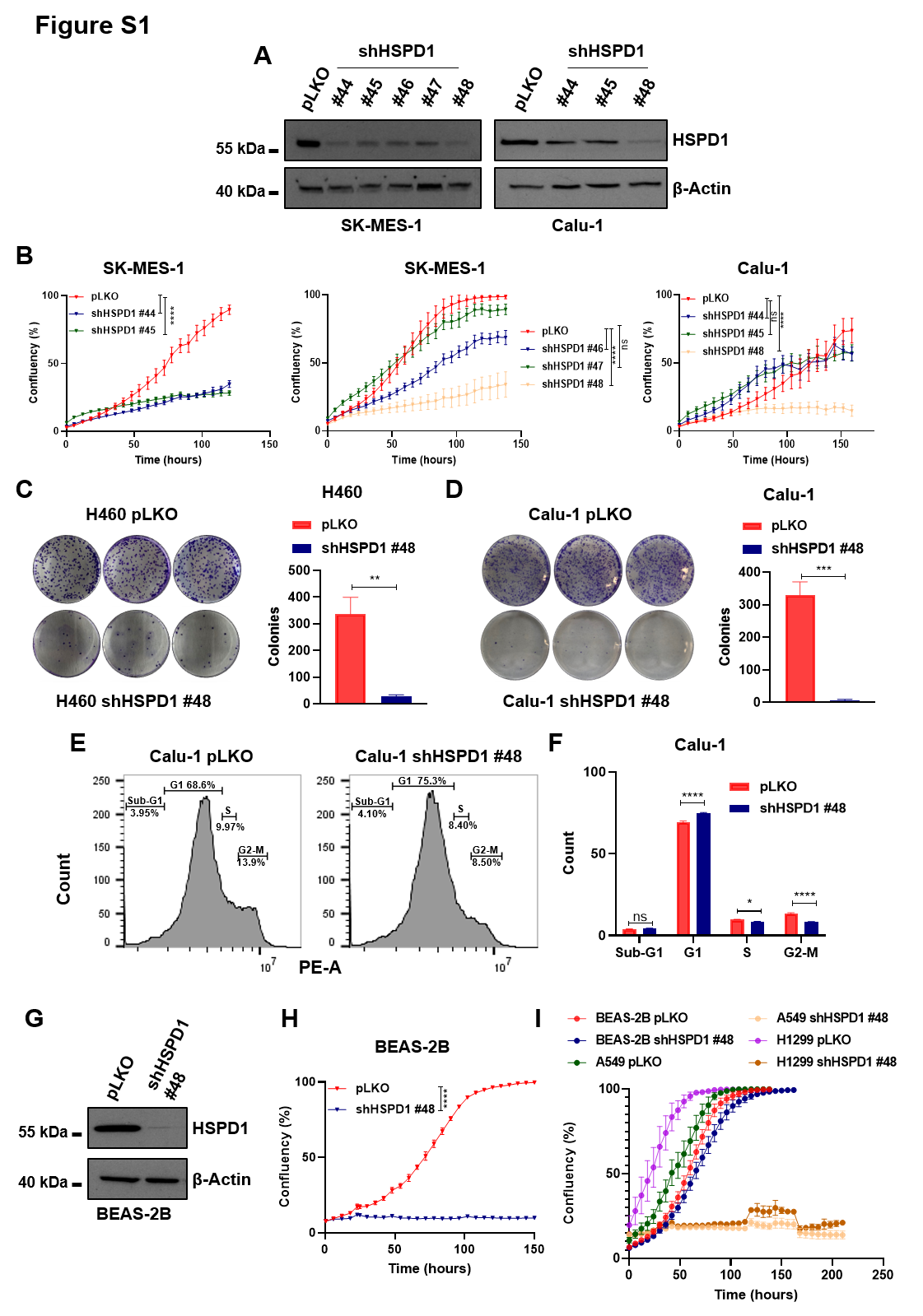


**Figure S2** A) Images of A549 and H1299 infected with pLKO or shHSPD1, stained with 400 nM MitoTracker Green one week after infection. B) Real-time proliferation curves of H23 and SK-MES-1 treated either with vehicle (DMSO) or KHS101. P-values are from two-way ANOVA. Points are average of biological replicates ± SD. ****<0.0001. C) Images of cells (A549, Calu-1, H1299, H460, H23 and SK-MES-1) treated with DMSO or KHS101 10 μM (after 5 days of treatment).


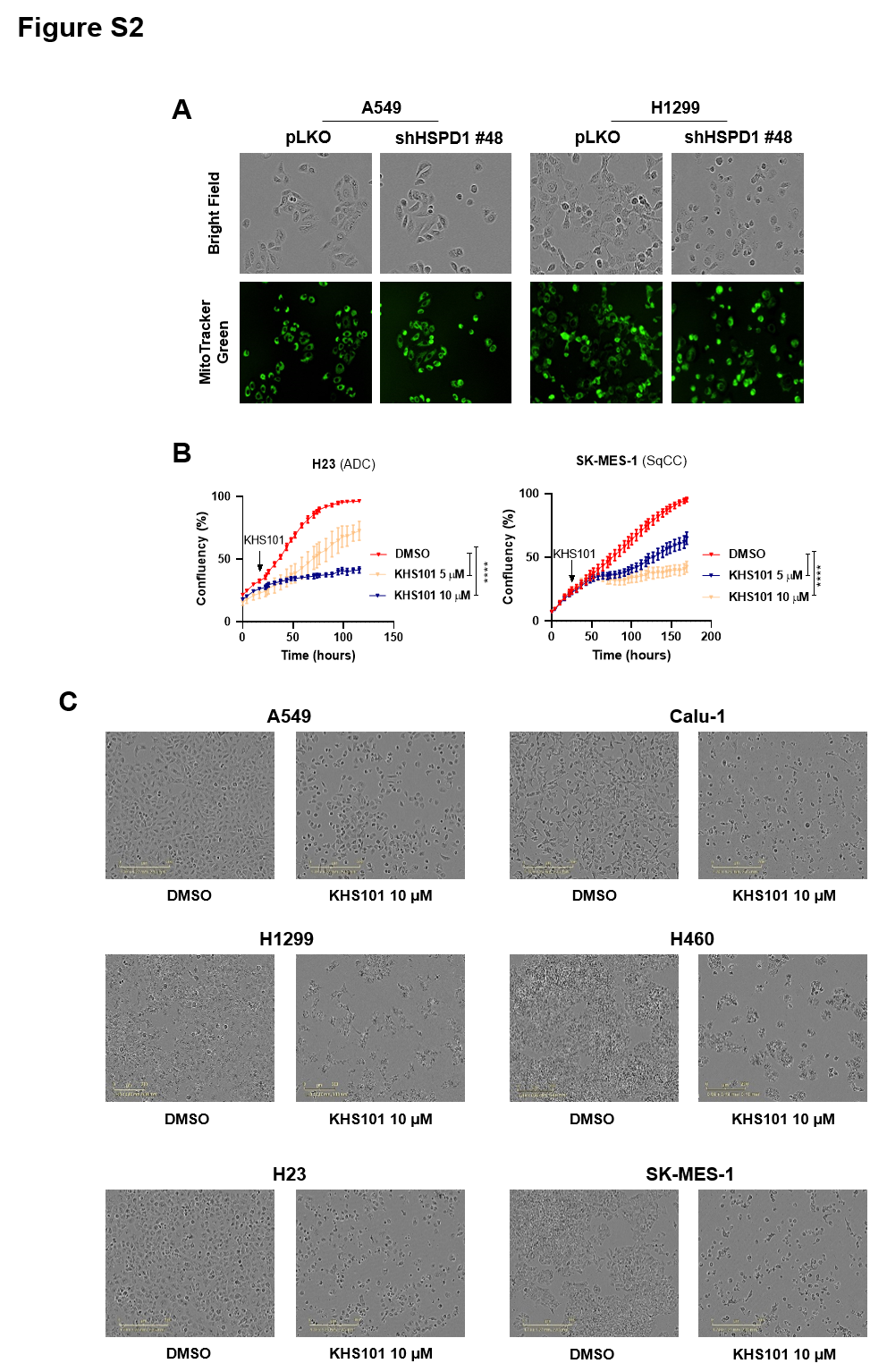


**Figure S3** A) Real-time proliferation curves of A549 cells treated either with KHS101 or with the corresponding inactive KHS101 analog (HB072) compared to vehicle-treated cells. Points are average values of biological replicates ± SD. P-values are from two-way ANOVA. *<0.05, ****<0.0001. B) Colony formation of Calu-1 treated for 5 days with KHS101 or vehicle and then left to grow in drug-free media, stained with crystal-violet and quantified in triplicates. Bars are average of biological replicates ± SD. P-values are from unpaired *t*-test. **<0.01. C) Real-time proliferation curves of A549 and Calu-1 treated with KHS101 in presence of different concentration of FBS in the media. Points are average of biological replicates ± SD.


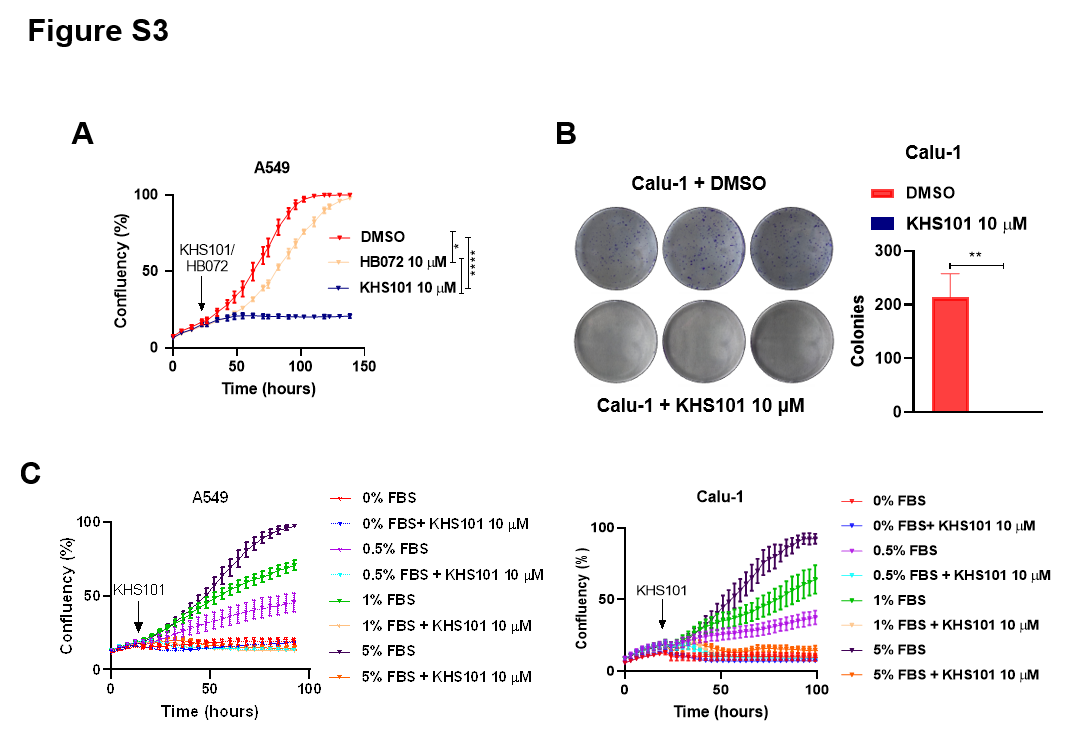


**Figure S4** Quantification of OCR (basal respiration) and ECAR (indicative glycolysis) of A549 (A) and Calu-1 (B) cells treated for 0.5 h, 2 h, 4 h and 6 h compared to control cells. Bars are average values of replicates ± SD. P-values are from unpaired *t*-test. *<0.05, ****<0.0001. C) qPCR quantification of relative mRNA levels of key metabolic enzymes HK2, FBP1 and PKM in A549 shHSPD1 #48 and A549 treated 72 hours with KHS101. Bars are average of biological replicates ± SD. P-values are from two-way ANOVA. *<0.05, **<0.01. D) Electron microscopy images of A549 treated either for 48 or 96 hours with KHS101 10 μM. L-lysosomes, M-mitochondria. Dead cell quantification as green object count using Cytotox green reagent (E) or caspse-3/7 reagent (F) of vehicle or KHS101 treated Calu-1 cells. G) Images of A549 and Calu-1 cells treated with vehicle or KHS101 and stained with caspase-3/7 reagent. H) Dead cell quantification as shown per green object count of Calu-1 treated with DMSO or KHS101 in combination with pan-caspase inhibitor Z-VAD-FMK. I) Dead cell quantification (green object count) using caspase3/7 green reagent of A549 treated either with DMSO or KHS101 in combination with pan caspase inhibitor Z-VAD-FMK. J) Dead cell quantification as per green object count of A549 cells treated with DMSO or KHS101 in combination with necroptosis inhibitor Necrostatin-1. K) Real-time proliferation curves of A549 cells treated either with DMSO or KHS101 in presence of ferroptosis inhibitor Ferrostatin-1. In E, F, H, I, J and K points are average values of replicates ± SD. P-values are from two-way ANOVA. **<0.01, ****<0.0001.


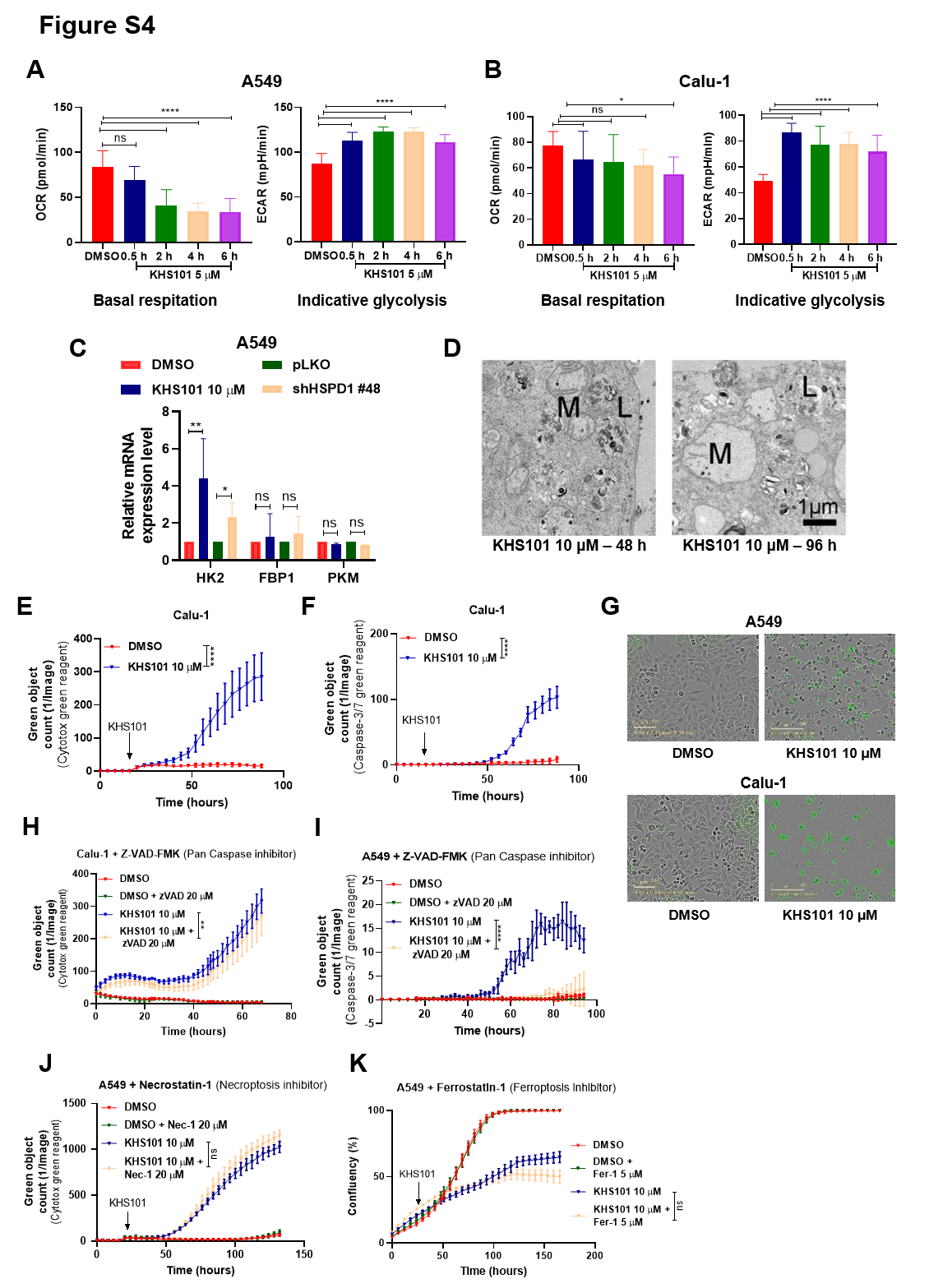


**Figure S5** A) Real-time proliferation curves of mouse cell lines LL2 treated either with vehicle (DMSO) or KHS101. Points are average of replicates ± SD. P-values are from two-way ANOVA. ****<0.0001. B) Images of LL2 treated with DMSO or KHS101 10 μM. C) Kaplan–Meier disease-specific survival analysis of C57BL/6 mice treated with 6 mg/kg KHS101 or vehicle (5% (v/v) Ethanol - 15% (w/v) Captisol solution) for 2 weeks. P-value based on Log-rank test. *<0.05. Disease-specific survival time (shown on the right panel) was calculated starting from the first appearance of bioluminescence. Bars are average of mice survival times ± SD. P-value based on unpaired *t*-test. *<0.05. D) Bar graph showing number of lung lesions (metastasis) of the KHS101 treated group compared to control. Bars are average of lesion numbers ± SD. P-value based on unpaired *t*-test.


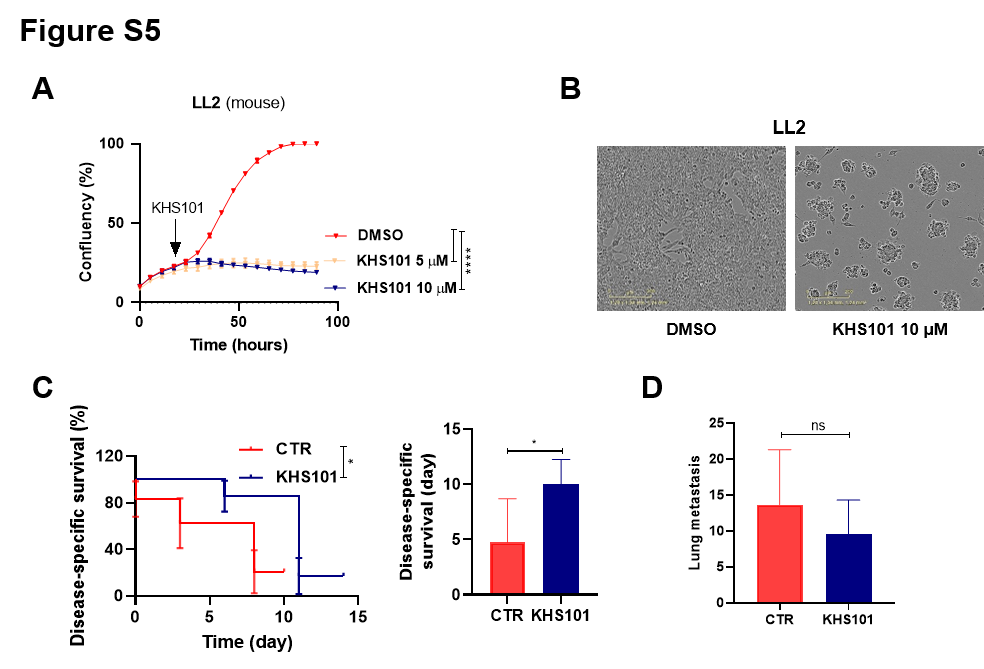


**Figure S6** Real-time proliferation curves (A) and dead cell quantification (B) of H1299 cells treated with KHS101 and/or cisplatin at indicated doses compared to control cells. Points are average of biological replicates ± SD. P-value are from two-way ANOVA and analyzed comparing the combination of drugs to control and the respective drug alone. ***<0.001, ****<0.0001.C) Dose-response curves to cisplatin of A549, H460 and H1299 cells treated with different concentration of KHS101. IC_50_ values (μM) are shown. Points are average values of replicates ± SD.


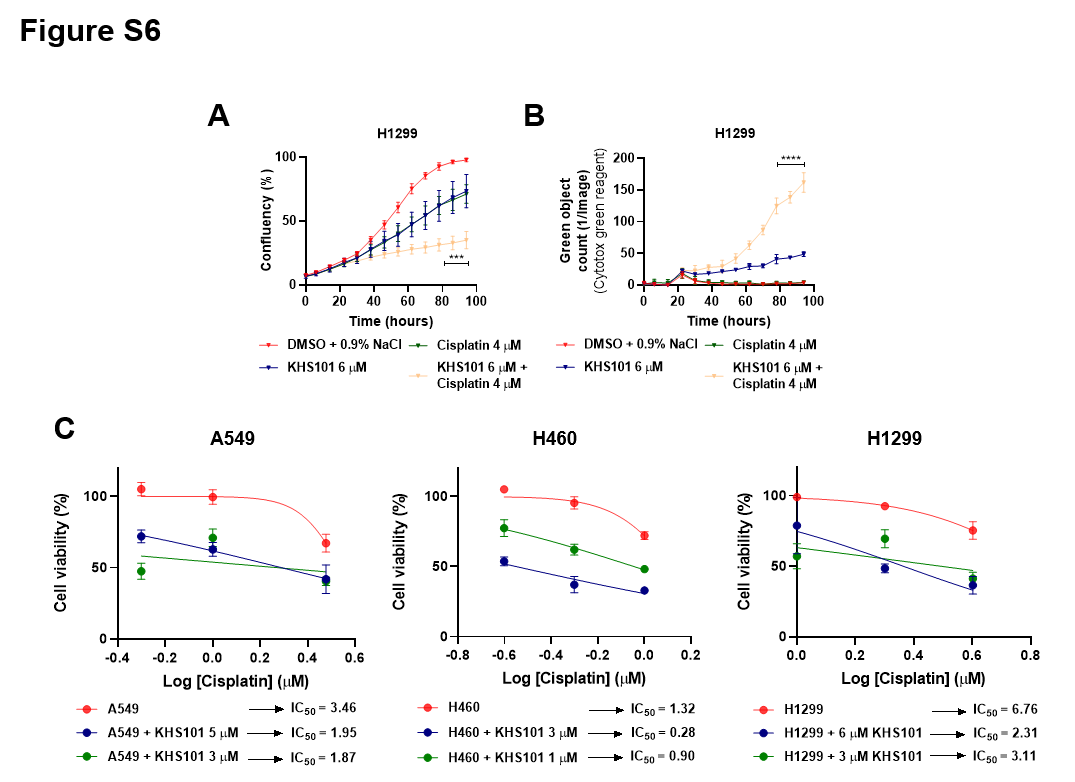


**Figure S7** A) Dose-response curves (normalized to DMSO control) to KHS101 of the remaining 24 NSCLC cell lines belonging to CL-100 ProLiFiler screening. Points are average values of replicates ± SD. IC_50_ values (μM) are shown with 95% confidence intervals.


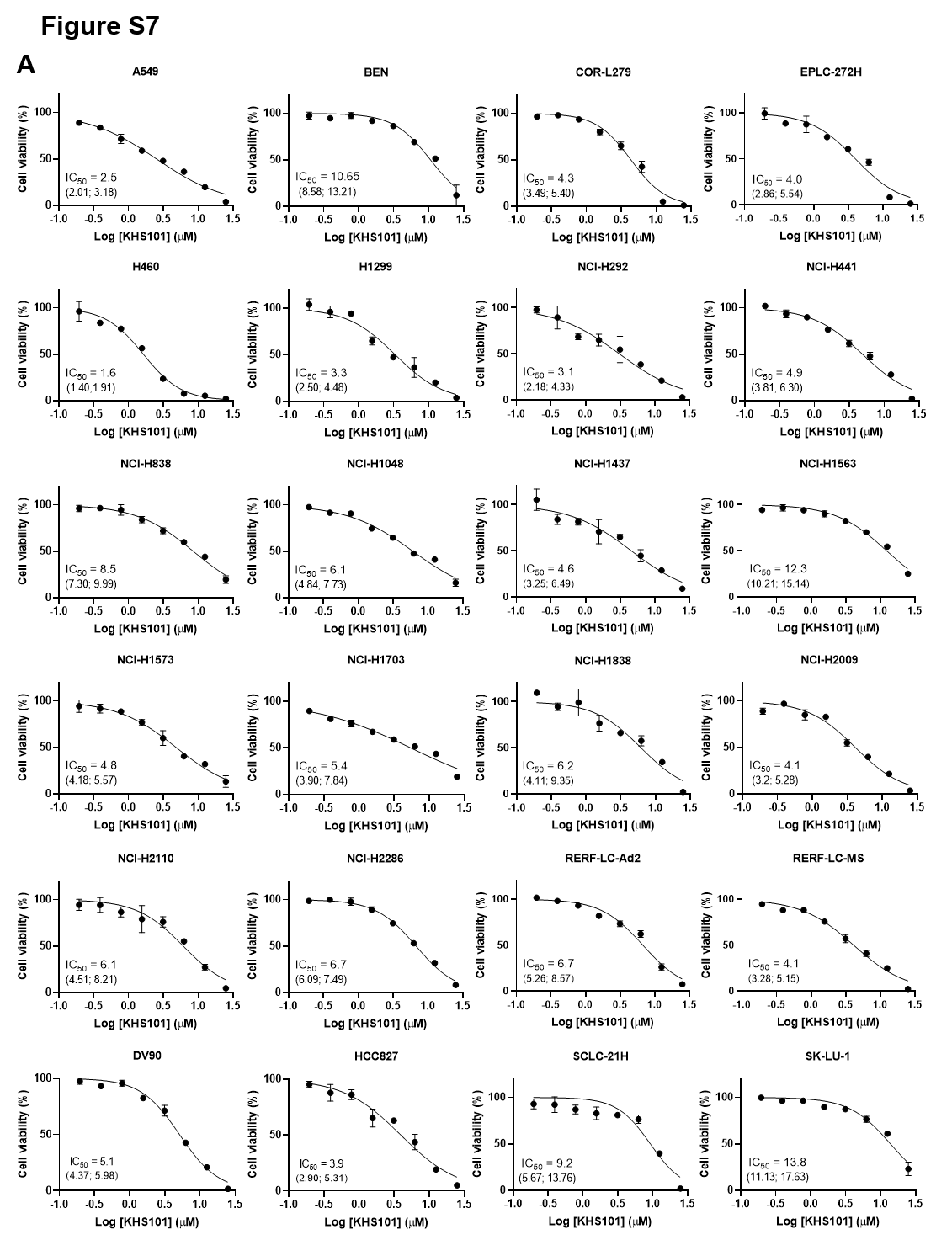


**Figure S8** A) Dose-response curves of 4 cell lines (H460, A549, NCI-H838 and BEN) as validation of the CL-100 ProLiFiler screening. Points are average of biological replicates ± SD. B) Venn diagrams showing overlap of up- or down-regulated genes in resistant cells identified in the transcriptome analysis and IC_50_ correlation analysis. C) Dose-response curves (normalized to DMSO control) of H1299 and H460 overexpressing NLRC5 compared to empty control cells. Points are average values of replicates ± SD. IC_50_ values (μM) are shown. P-values are from two-way ANOVA. **<0.01.


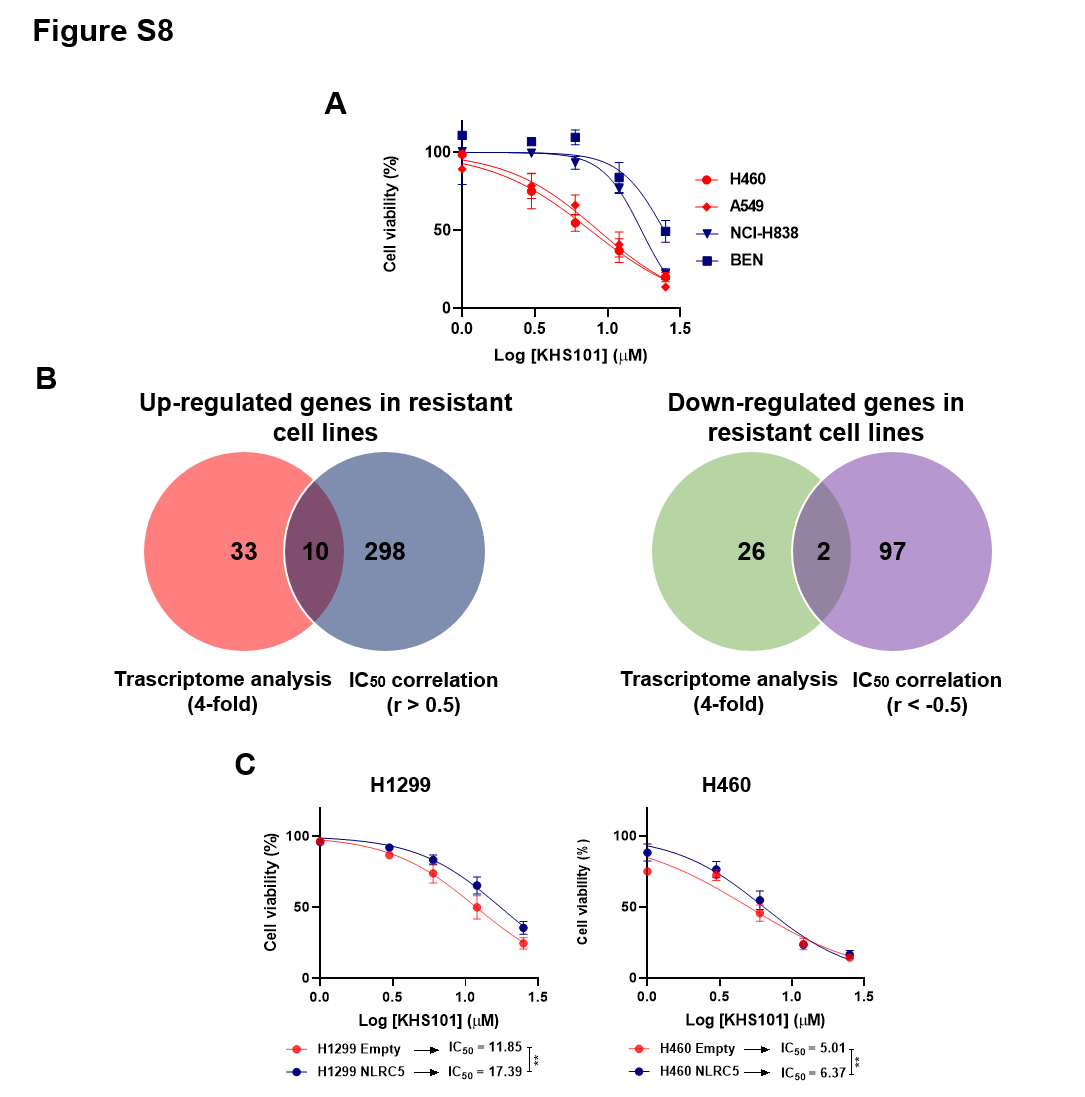


**Table S1**

| **CL-100 ProLiFiler screening** | |
| --- | --- |
| **Cell line** | **Medium** |
| A549 | DMEM + 10% FCS |
| BEN | DMEM + 10% FCS |
| COR-L279 | RPMI-1640+10% FCS |
| DV90 | RPMI-1640 + 10% FCS |
| EPLC-272H | RPMI-1640+10% FCS |
| H1299 | DMEM + 10% FCS |
| H460 | DMEM + 10% FCS |
| HCC827 | RPMI-1640+10% FCS |
| LOU-NH91 | RPMI-1640+10% FCS |
| NCI-H1048 | RPMI-1640 + 10% FCS |
| NCI-H1437 | RPMI-1640 + 10% FCS |
| NCI-H1563 | RPMI-1640+10% FCS |
| NCI-H1573 | RPMI-1640+10% FCS |
| NCI-H1581 | RPMI-1640 + 10% FCS |
| NCI-H1703 | RPMI-1640 + 10% FCS |
| NCI-H1838 | RPMI-1640+10% FCS |
| NCI-H2009 | RPMI-1640+10% FCS |
| NCI-H2110 | RPMI-1640 + 10% FCS |
| NCI-H2286 | RPMI-1640+10% FCS |
| NCI-H292 | RPMI-1640+10% FCS |
| NCI-H441 | RPMI-1640+10% FCS |
| NCI-H838 | RPMI-1640 + 10% FCS |
| RERF-LC-Ad2 | RPMI-1640+10% FCS |
| RERF-LC-MS | RPMI-1640 + 10% FCS |
| SCLC-21H | DMEM + 10% FCS |
| SK-LU-1 | DMEM + 10% FCS |

**Table S2**

| **HSPD1 sensitivity score from PROJECTDRIVE** | |
| --- | --- |
| **Cell line** | **Sensitivity Score** |
| NCIH1568 | -11.94 |
| NCIH1944 | -11.06 |
| CALU6 | -10.19 |
| NCIH1437 | -10.18 |
| NCIH1299 | -9.45 |
| NCIH2172 | -9.44 |
| NCIH1355 | -8.68 |
| NCIH23 | -8.61 |
| HCC15 | -8.45 |
| NCIH1793 | -8.29 |
| KNS62 | -7.59 |
| HCC1359 | -6.82 |
| NCIH441 | -6.63 |
| NCIH358 | -6.52 |
| EBC1 | -6.44 |
| NCIH1703 | -6.15 |
| ABC1 | -6.10 |
| NCIH2030 | -6.03 |
| CORL23 | -5.76 |
| RERFLCMS | -5.65 |
| HCC4006 | -5.25 |
| NCIH2110 | -5.18 |
| A549 | -4.47 |
| SQ1 | -4.40 |
| NCIH522 | -4.20 |
| HCC44 | -4.17 |
| NCIH2170 | -4.16 |
| NCIH1693 | -4.02 |
| NCIH661 | -4.01 |
| NCIH1792 | -3.97 |
| NCIH1373 | -3.74 |
| NCIH460 | -3.66 |
| NCIH2122 | -3.64 |
| NCIH2009 | -3.63 |
| LCLC103H | -3.63 |
| SW1573 | -3.43 |
| NCIH1975 | -3.23 |

**Table S3**

| **Mutation profile of the top 4 more sensitive and resistant cell lines obtained in the screening** | | | | | | | | |
| --- | --- | --- | --- | --- | --- | --- | --- | --- |
| **Gene** | **NCI-H460** | **NCI-H1581** | **LOU-H91** | **A549** | **SK-LU-1** | **NCI-H1563** | **BEN** | **NCI-H838** |
| TP53 | NM | NS (p.Q144*) | MS (p.V143M) | NM | MS (p.H193R) | NM | MS (p.Y163C) | NS (p.E62*) |
| PIK3CA | MS (p.E545K) | NM | MS (p.E726K) | NM | NM | MS (p.E542K) | MS (p.G967R) | NM |
| KRAS | MS (p.Q61H) | NM | NM | MS (p.G12S) | MS (p.G12D) | NM | NM | NM |
| STK11 | NS (p.Q37*) | NM | NM | NS (p.Q37*) | NM | MS (p.G242W), NS (p.Y272*) | NM | NM |

NM = no mutation

MS = missense

NS = nonsense

**Table S4**

| **4-Fold up-regulated genes in resistant cell lines** | | |
| --- | --- | --- |
| **Genes** | **logFC** | **p-value** |
| CPE | 5.01 | 0.0033 |
| ACKR3 | 4.13 | 0.0073 |
| GRAMD3 | 3.97 | 0.0003 |
| HCP5 | 3.74 | 0.0289 |
| FOS | 3.47 | 0.0257 |
| NDRG1 | 3.04 | 0.0053 |
| CARD16 | 2.96 | 0.0271 |
| HLA-F | 2.93 | 0.0332 |
| PSMB8-AS1 | 2.82 | 0.0346 |
| ARMCX1 | 2.82 | 0.0389 |
| SATB1 | 2.70 | 0.0231 |
| LNX1 | 2.68 | 0.0350 |
| TMTC1 | 2.60 | 0.0075 |
| MCOLN3 | 2.59 | 0.0035 |
| PLPP3 | 2.57 | 0.0163 |
| TNFRSF21 | 2.57 | 0.0219 |
| PPARGC1A | 2.53 | 0.0493 |
| MSC-AS1 | 2.48 | 0.0466 |
| NLRC5 | 2.43 | 0.0052 |
| TMCC3 | 2.41 | 0.0083 |
| TNFSF10 | 2.37 | 0.0427 |
| UST | 2.36 | 0.0109 |
| DNAJA4 | 2.35 | 0.0491 |
| NMNAT2 | 2.31 | 0.0233 |
| LINC00648 | 2.25 | 0.0112 |
| HLA-J | 2.24 | 0.0250 |
| TAPBPL | 2.24 | 0.0237 |
| TLE2 | 2.21 | 0.0394 |
| ST3GAL6 | 2.14 | 0.0340 |
| AK4 | 2.12 | 0.0500 |
| PRSS21 | 2.08 | 0.0352 |
| HLA-DMA | 2.07 | 0.0487 |
| NDNF | 2.05 | 0.0324 |

**Table S5**

| **4-Fold down-regulated genes in resistant cell lines** | | |
| --- | --- | --- |
| **Genes** | **logFC** | **p-value** |
| SH3KBP1 | -2.11 | 0.0186 |
| C11ORF70 | -2.13 | 0.0329 |
| TGFB2 | -2.15 | 0.0373 |
| ECHDC3 | -2.17 | 0.0144 |
| SLFN11 | -2.22 | 0.0443 |
| HHEX | -2.22 | 0.0003 |
| MTAP | -2.23 | 0.0127 |
| GSAP | -2.24 | 0.0304 |
| FOXF2 | -2.31 | 0.0195 |
| SNHG5 | -2.37 | 0.0004 |
| HIST1H1D | -2.39 | 0.0063 |
| FAM133A | -2.48 | 0.0470 |
| RFLNB | -2.49 | 0.0144 |
| NRXN3 | -2.53 | 0.0223 |
| CEP112 | -2.53 | 0.0404 |
| SMKR1 | -2.55 | 0.0057 |
| P2RX5 | -2.57 | 0.0083 |
| SLC6A8 | -2.61 | 0.0151 |
| SEMA3A | -2.84 | 0.0080 |
| GLB1L2 | -3.02 | 0.0031 |
| EEF1A2 | -3.23 | 0.0356 |
| LDOC1 | -3.57 | 0.0231 |
| CDH11 | -4.39 | 0.0208 |
| VIM | -4.56 | 0.0477 |
| COL5A2 | -5.54 | 0.0120 |
| GJA1 | -7.11 | 0.0001 |

**Table S6**

| **Positively correlated genes with IC50 values (r>0.5)** | |
| --- | --- |
| **Genes** | **Correlation** |
| RNF180 | 0.791 |
| BTD | 0.790 |
| NLRC5 | 0.777 |
| PSMB8-AS1 | 0.766 |
| GVINP1 | 0.759 |
| TAPBPL | 0.754 |
| PPP1R36 | 0.741 |
| HLA-E | 0.738 |
| ATP8A2 | 0.730 |
| SNTB1 | 0.723 |
| FRS2 | 0.722 |
| HLA-J | 0.718 |
| NPY1R | 0.716 |
| LVCAT1 | 0.713 |
| BEND5 | 0.709 |
| RGS4 | 0.707 |
| HLA-F | 0.707 |
| HLA-B | 0.702 |
| MFSD7 | 0.693 |
| PSMB8 | 0.692 |
| STBD1 | 0.692 |
| HCG26 | 0.691 |
| MME | 0.689 |
| LINC01268 | 0.685 |
| HCP5 | 0.684 |
| ACSL5 | 0.683 |
| SH3RF3 | 0.682 |
| fam149a | 0.680 |
| MAB21L2 | 0.676 |
| TG | 0.675 |
| HLA-C | 0.675 |
| BIN1 | 0.672 |
| SLC25A53 | 0.672 |
| RCAN1 | 0.672 |
| CASP1 | 0.671 |
| CTSO | 0.670 |
| SERPINA2 | 0.669 |
| NUDT9 | 0.665 |
| NINJ2 | 0.658 |
| LY96 | 0.658 |
| LINC01082 | 0.657 |
| ETV2 | 0.655 |
| SPTLC3 | 0.649 |
| IFITM10 | 0.648 |
| BTN3A3 | 0.645 |
| B2M | 0.642 |
| BTN3A2 | 0.640 |
| GUCY1A2 | 0.639 |
| HCFC1R1 | 0.636 |
| DPYSL2 | 0.636 |
| GBP1P1 | 0.636 |
| DAO | 0.634 |
| GAS5-AS1 | 0.633 |
| TMEM184C | 0.633 |
| EVC | 0.633 |
| PLPP3 | 0.631 |
| MYL2 | 0.631 |
| PRR32 | 0.628 |
| LOC100507642 | 0.626 |
| PARP3 | 0.622 |
| TMEM229B | 0.618 |
| TRPC3 | 0.618 |
| TPP1 | 0.618 |
| ANXA5 | 0.617 |
| CTSD | 0.617 |
| GPR108 | 0.617 |
| CSF1R | 0.614 |
| ptger3 | 0.613 |
| CDH26 | 0.613 |
| ADAMTS15 | 0.610 |
| CASP3 | 0.607 |
| TMEM99 | 0.605 |
| TBXAS1 | 0.603 |
| ATP10D | 0.603 |
| TCN2 | 0.602 |
| PLA2G1B | 0.602 |
| MEGF8 | 0.602 |
| FLT3LG | 0.601 |
| PRKAG3 | 0.601 |
| WSB1 | 0.600 |
| nlgn1 | 0.600 |
| MMAA | 0.599 |
| WIPI1 | 0.599 |
| ZCCHC5 | 0.598 |
| C14orf37 | 0.598 |
| PRDM5 | 0.597 |
| SNX25 | 0.597 |
| MAGEB6 | 0.597 |
| HM13 | 0.596 |
| SSPN | 0.596 |
| RIMS1 | 0.595 |
| LOC100049716 | 0.594 |
| GABRG2 | 0.593 |
| GBP4 | 0.591 |
| JAM2 | 0.590 |
| CFAP52 | 0.589 |
| CCDC130 | 0.588 |
| HLA-DPB1 | 0.588 |
| ZNF75A | 0.587 |
| BMP2K | 0.587 |
| AKAP14 | 0.583 |
| MBNL1-AS1 | 0.583 |
| KCNN2 | 0.582 |
| NTNG2 | 0.582 |
| plxdc2 | 0.582 |
| NAV1 | 0.581 |
| HELQ | 0.581 |
| hla-dqa1 | 0.580 |
| PLSCR4 | 0.579 |
| NOBOX | 0.579 |
| NDN | 0.578 |
| LPXN | 0.577 |
| CRADD | 0.577 |
| CHST2 | 0.576 |
| IFT46 | 0.576 |
| WISP1 | 0.576 |
| CEBPE | 0.574 |
| PIH1D1 | 0.574 |
| MYBPH | 0.573 |
| ARHGEF6 | 0.572 |
| SYNC | 0.572 |
| CCDC96 | 0.572 |
| CXCL10 | 0.571 |
| RRAGB | 0.570 |
| GRN | 0.570 |
| BTBD3 | 0.570 |
| AES | 0.569 |
| UBOX5 | 0.569 |
| BHLHE23 | 0.569 |
| NDST3 | 0.568 |
| COL6A3 | 0.566 |
| IL12RB1 | 0.566 |
| STEAP3 | 0.566 |
| WDFY3 | 0.566 |
| ZBTB47 | 0.565 |
| SURF1 | 0.564 |
| SLC25A4 | 0.564 |
| Trac | 0.563 |
| SP6 | 0.563 |
| CD1D | 0.562 |
| LINC00458 | 0.562 |
| ZNF311 | 0.561 |
| MOK | 0.561 |
| IGFBP5 | 0.560 |
| BEX5 | 0.560 |
| CR1L | 0.559 |
| OLFML2B | 0.559 |
| SHISA3 | 0.558 |
| ZNF629 | 0.557 |
| MTTP | 0.557 |
| TLR3 | 0.557 |
| STX18-AS1 | 0.556 |
| arfip1 | 0.556 |
| LPAR4 | 0.555 |
| hla-a | 0.555 |
| MAPK8IP3 | 0.555 |
| CXCL11 | 0.555 |
| CHMP4B | 0.554 |
| GPR68 | 0.554 |
| NCALD | 0.553 |
| RUNDC3B | 0.552 |
| KIAA1644 | 0.552 |
| ADAM21 | 0.551 |
| EMSY | 0.551 |
| CYCS | 0.551 |
| OAS2 | 0.551 |
| IRF1 | 0.550 |
| BCL2L1 | 0.550 |
| WEE2-AS1 | 0.550 |
| H2AFV | 0.550 |
| CCRL2 | 0.549 |
| TMEM74B | 0.549 |
| BCAR1 | 0.548 |
| EDNRB | 0.548 |
| COL6A1 | 0.548 |
| LOC440028 | 0.548 |
| CYTH2 | 0.548 |
| ZCCHC18 | 0.547 |
| STX5 | 0.545 |
| LOXL4 | 0.545 |
| OR8B8 | 0.545 |
| MYL5 | 0.543 |
| GRM8 | 0.541 |
| PTN | 0.541 |
| MOCS1 | 0.541 |
| KLF12 | 0.541 |
| cyp2u1 | 0.540 |
| COL1A1 | 0.540 |
| MAP3K7CL | 0.540 |
| SLC15A3 | 0.539 |
| RABAC1 | 0.539 |
| GMPR | 0.539 |
| KDM4B | 0.538 |
| C1QTNF2 | 0.538 |
| MROH8 | 0.536 |
| CDK15 | 0.536 |
| WNT5B | 0.535 |
| REG1CP | 0.535 |
| TRIM55 | 0.535 |
| hla-dra | 0.534 |
| MSX1 | 0.534 |
| MAP3K11 | 0.534 |
| DNAJB14 | 0.533 |
| STOML1 | 0.533 |
| PLCL1 | 0.533 |
| IQCF4 | 0.533 |
| LOXL1 | 0.532 |
| uba7 | 0.532 |
| SLC2A2 | 0.532 |
| ZFP91 | 0.531 |
| BCAR3 | 0.531 |
| ZNF215 | 0.531 |
| apol3 | 0.530 |
| C6orf226 | 0.530 |
| LGALS3BP | 0.530 |
| GAS1 | 0.529 |
| MAP4K4 | 0.529 |
| ERC2-IT1 | 0.529 |
| CAGE1 | 0.529 |
| NKX6-1 | 0.528 |
| SLC36A4 | 0.528 |
| LRRC56 | 0.528 |
| CAPN6 | 0.526 |
| SAMD9L | 0.525 |
| SIX2 | 0.525 |
| CWC25 | 0.525 |
| ACY3 | 0.524 |
| TREML3P | 0.524 |
| DIRAS2 | 0.524 |
| MXD4 | 0.522 |
| BTN3A1 | 0.522 |
| CYP8B1 | 0.522 |
| ACVR2A | 0.521 |
| HLA-DRB1 | 0.521 |
| ADAMTSL1 | 0.521 |
| LINC00639 | 0.520 |
| PTX3 | 0.520 |
| TGM2 | 0.519 |
| fam134b | 0.519 |
| MEIS3 | 0.519 |
| CAMK4 | 0.518 |
| zbtb3 | 0.518 |
| TEF | 0.518 |
| CLMP | 0.518 |
| RALGAPB | 0.517 |
| CCDC159 | 0.517 |
| AP5S1 | 0.517 |
| MS4A14 | 0.516 |
| LINC00852 | 0.516 |
| c19orf70 | 0.516 |
| SCARB2 | 0.516 |
| FKBP2 | 0.515 |
| ZNF704 | 0.515 |
| PLXNC1 | 0.515 |
| ATP2B2 | 0.515 |
| CHRNB2 | 0.514 |
| SNHG11 | 0.513 |
| CNOT6L | 0.513 |
| BDH2 | 0.512 |
| CD74 | 0.512 |
| GFI1 | 0.512 |
| MED16 | 0.512 |
| FAM43A | 0.511 |
| MXD3 | 0.511 |
| ZNF48 | 0.511 |
| GNA11 | 0.510 |
| TLE2 | 0.510 |
| KDM2A | 0.510 |
| ARL9 | 0.509 |
| ABCA5 | 0.509 |
| ORMDL3 | 0.508 |
| GAS6 | 0.507 |
| C10orf71 | 0.507 |
| TMUB2 | 0.507 |
| SLC5A12 | 0.506 |
| ITGA10 | 0.506 |
| EPB41L4A-AS2 | 0.506 |
| SLC39A13 | 0.506 |
| CBLN3 | 0.506 |
| HERPUD1 | 0.505 |
| VEGFB | 0.505 |
| PSMB9 | 0.504 |
| IZUMO4 | 0.504 |
| CUEDC1 | 0.504 |
| CFB | 0.504 |
| RNASE11 | 0.503 |
| LINC00969 | 0.503 |
| MCOLN3 | 0.502 |
| MARCO | 0.502 |
| CCDC93 | 0.502 |
| DCTD | 0.501 |
| LRRC66 | 0.501 |
| FIBP | 0.501 |
| KAT5 | 0.501 |
| C10orf113 | 0.501 |
| NMNAT2 | 0.500 |
| LOC283038 | 0.500 |
| PAX5 | 0.500 |

**Table S7**

| **Negatively correlated genes with IC50 values (r<-0.5)** | |
| --- | --- |
| **Genes** | **Correlation** |
| ANKS6 | -0.746 |
| GLRX3 | -0.741 |
| RPL13 | -0.698 |
| FAM206A | -0.683 |
| LYNX1 | -0.672 |
| WBSCR22 | -0.662 |
| LOC440704 | -0.660 |
| CTU2 | -0.659 |
| c10orf76 | -0.657 |
| DUS4L | -0.641 |
| PPA1 | -0.627 |
| FN3K | -0.623 |
| POLA1 | -0.621 |
| EIF3A | -0.615 |
| MRPL43 | -0.615 |
| POLR2A | -0.608 |
| SLC6A8 | -0.604 |
| NKD1 | -0.603 |
| EIF1AX | -0.602 |
| armc9 | -0.598 |
| ZCCHC9 | -0.597 |
| IKBKAP | -0.593 |
| RXFP1 | -0.589 |
| PUDP | -0.589 |
| GBF1 | -0.583 |
| NOLC1 | -0.582 |
| SHH | -0.575 |
| ADPGK | -0.575 |
| DUSP22 | -0.575 |
| HAND1 | -0.571 |
| PGAM5 | -0.571 |
| hyal3 | -0.565 |
| HTR1A | -0.564 |
| LINC00544 | -0.564 |
| SLC15A4 | -0.563 |
| LDLRAP1 | -0.563 |
| ANKLE1 | -0.563 |
| RRP9 | -0.562 |
| aire | -0.559 |
| pelp1 | -0.558 |
| COG5 | -0.555 |
| zfp64 | -0.554 |
| RRP12 | -0.553 |
| ACP1 | -0.553 |
| RHBDF2 | -0.552 |
| RIT2 | -0.551 |
| ARMC10 | -0.549 |
| MRGPRG-AS1 | -0.545 |
| SMYD5 | -0.544 |
| EIF2S3 | -0.542 |
| SIGMAR1 | -0.541 |
| USP54 | -0.540 |
| ALDH18A1 | -0.539 |
| NUP93 | -0.539 |
| PDC | -0.539 |
| DHODH | -0.537 |
| HPS6 | -0.536 |
| TCOF1 | -0.536 |
| TNFRSF4 | -0.535 |
| BCCIP | -0.534 |
| TRAV12-1 | -0.533 |
| PES1 | -0.533 |
| HSD17B3 | -0.532 |
| PAPOLB | -0.532 |
| dhx37 | -0.529 |
| NEUROG3 | -0.529 |
| TFB1M | -0.529 |
| SMC1A | -0.527 |
| NME1 | -0.527 |
| RSG1 | -0.527 |
| BLOC1S2 | -0.521 |
| P2RX5 | -0.521 |
| C1QBP | -0.519 |
| ODF1 | -0.518 |
| ttll13p | -0.518 |
| BTNL2 | -0.517 |
| NUP88 | -0.516 |
| MRM1 | -0.515 |
| IQCK | -0.515 |
| pprc1 | -0.514 |
| CD163 | -0.512 |
| IARS | -0.511 |
| rpp25 | -0.510 |
| DDX51 | -0.510 |
| OGFOD1 | -0.510 |
| SLC25A13 | -0.508 |
| ARID1A | -0.507 |
| ZDHHC16 | -0.507 |
| C12ORF10 | -0.507 |
| INPP5A | -0.507 |
| NOC4L | -0.507 |
| LOC100506405 | -0.506 |
| RPL38 | -0.504 |
| CHRNA1 | -0.504 |
| DIMT1 | -0.504 |
| EXOSC1 | -0.503 |
| PDCD11 | -0.501 |

**Table S8**

| **Top 20 genes in CRISPR/Cas9 screening** | |
| --- | --- |
| **Gene** | **Pos-score** |
| COX5B | 0.000016396 |
| MARK4 | 0.000025227 |
| TATDN3 | 0.000030289 |
| CTAGE1 | 0.000040364 |
| CBR1 | 0.000044941 |
| hsa-mir-365a | 0.000051036 |
| TMC8 | 0.000056552 |
| hsa-mir-138-1 | 0.000066093 |
| SLC25A52 | 0.000070277 |
| CCDC106 | 0.000075681 |
| STK39 | 0.000077296 |
| hsa-mir-181d | 0.000096834 |
| hsa-mir-6856 | 0.00011253 |
| hsa-mir-7850 | 0.00011772 |
| LIPG | 0.00012613 |
| hsa-mir-6730 | 0.00015096 |
| hsa-mir-1184-3 | 0.00015597 |
| hsa-mir-548f-5 | 0.00018152 |
| ACSL5 | 0.00020533 |
| PDE2A | 0.00021423 |
